# Prevalence and Geographical Distribution of Foodborne *Yersinia enterocolitica* in Chinese Livestock and Their Products: A Systematic Review and Meta-Analysis (2000–2024)

**DOI:** 10.3390/ani16030418

**Published:** 2026-01-29

**Authors:** Wen-Bo Lou, Ran Zhao, Siddique Sehrish, Yu-Hao Song, Qing-Long Gong, Rui Du

**Affiliations:** 1 College of Veterinary Medicine, Jilin Agricultural University, Changchun 130118, China; l2368264168@163.com (W.-B.L.); rzhao390@gmail.com (R.Z.); sehrishsiddique2017@gmail.com (S.S.); yuhao.song@mails.jlau.edu.cn (Y.-H.S.); 2 Jilin Provincial Engineering Research Center for Sika Deer Efficient Breeding and Product Development Technology, Jilin Agricultural University, Changchun 130118, China; 3 Department of Animal Medicine, College of Agriculture, Yanbian University, Yanji 133000, China

**Keywords:** *Y. enterocolitica*, zoonosis, prevalence, food safety, meat, China

## Abstract

*Yersinia enterocolitica* is a psychrotrophic zoonotic pathogen that causes diarrhea in animals and enteritis in humans, mainly transmitted through the food chain. This systematic review and meta-analysis estimated the prevalence, geographical distribution, and related risk factors of *Y. enterocolitica* in livestock throughout the Chinese Mainland. Comprehensive searches were conducted in PubMed, ScienceDirect, CNKI, Wanfang, and VIP databases for studies between 1 January 2000 and 1 August 2025. Out of 1092 identified studies, 28 met the inclusion criteria. The estimated overall prevalence of *Y. enterocolitica* was 9.37%. Prior to 2015, the prevalence peaked at 9.69% but declined in subsequent years. The highest prevalence was found in Southern China (25.00%). Among livestock species, pigs showed higher susceptibility (9.93%) compared to cattle (4.67%). Meat samples exhibited the highest prevalence (15.47%), while qPCR yielded the highest detection rate (10.79%). Geographical factors such as longitude, latitude, altitude, climate, temperature, rainfall, and humidity also influenced prevalence patterns. *Y. enterocolitica* remains widely distributed in livestock and meat products. Variability was linked to regional, species-specific, and methodological aspects, highlighting the need for One-Health-based monitoring, stricter hygiene regulations, and standardized diagnostics to protect food safety.

## Simple Summary

*Y. enterocolitica* is a psychrotrophic zoonotic bacterium transmitted through contaminated animal products, yet no long-term national synthesis of its prevalence in Chinese livestock has previously been reported. This meta-analysis and systematic review of peer-reviewed research articles published between 2000 and 1 August 2025; we screened 1092 records and included 28 studies covering 5842 animals across 15 provinces. The pooled prevalence was 9.37% (5.55–14.03), with significant geographical variations, including the largest burden in Southern China, and higher rates in studies conducted before 2015. Pigs had the highest prevalence rate (≈10%) while cattle, sheep, and goats had a lower one (<5%). Regarding detection sensitivity, qPCR was more sensitive than culture-based techniques, and meat samples yielded higher detection rates than fecal samples. Univariate meta-regression showed that pathogen occurrence was positively correlated with temperature, rainfall, altitude, and humidity. Overall, these findings showed that *Y. enterocolitica* remains widespread in Chinese livestock and meat products, highlighting the need for adopting sensitive, standardized diagnostics within a One-Health framework, enhancing slaughterhouse hygiene, and implementing region-specific biosecurity measures.

## 1. Introduction

A number of pathogens, including *Y. enterocolitica*, a facultative anaerobic Gram-negative bacillus, have been associated with contamination in water, food, dairy products, and ready-to-eat items sold in retail outlets, street markets, and supermarkets. Yersiniosis is a major foodborne zoonosis with widespread global distribution [1,2]. Its widespread distribution in nature and broad range of animal hosts facilitate transmission through the fecal oral route, causing illness ranging from enteritis and lymphadenitis to septicemia in both animals and humans [3]. Beyond its public health threat, *Y. enterocolitica* infects main livestock species, including pigs, cattle, sheep, and goats, leading to diarrhea, growth retardation, and mortality in young animals. This not only impacts animals’ welfare but also causes substantial economic losses in the livestock industry through meat contamination and trade restrictions [4,5].

In recent years, yersiniosis has gained recognition as a major global foodborne zoonosis. Its increasing incidence in Europe, North America, and Asia has led the World Health Organization (WHO) to designate it as a major concern for surveillance [6,7]. Within Europe, *Y. enterocolitica* ranks as the fourth most commonly identified bacterial food-borne pathogen after *Campylobacter* sp., *Salmonella* sp., and *Escherichia coli O157*, accounting for more than 7000 cases each year [8]. This pathogen’s zoonotic cycle is maintained in food-producing animals, with pigs identified as the key reservoir. Persistent colonization of pig tonsils and intestines leads to contamination of pork during processing [9,10]. While cattle, sheep, and goats are less common hosts with generally low prevalence, specifically regional and husbandry factors can amplify their contribution to the overall epidemiological risk [11,12].

Globally, *Y. enterocolitica* contamination remains a significant concern, characterized by high pathogen isolation rates and the establishment of antimicrobial-resistant strains, suggesting a threat that may impact the broader food chain [13]. In pig farms, especially in regions like Siberia, the bacterial persistence is directly linked to specific environmental and husbandry management, a pattern that aligns with regional distribution trends identified in China [14]. Moreover, methodological factors, specifically the integration of molecular tools with culture techniques, increase in detection sensitivity and influence prevalence estimates [15]. Similarly, the detection of *Y. enterocolitica* in humans and animals across Japan, Korea, and Thailand confirmed its public health significance in certain regions of Asia, across diverse livestock systems and dietary practices [16]. Nowadays, emerging evidence suggests that, beyond pork, *Y. enterocolitica* may also be associated with other food vehicles [17]. However, in many developing countries, *Y. enterocolitica* is closely monitored, yet diagnostic procedures remain limited in several developing regions, including China [18,19].

As the world’s leading pork and a major livestock producer, China may face a proportionally higher risk of exposure and transmission. Isolations of the pathogen from fecal samples of pigs, cattle, sheep, and goats have been documented in multiple Chinese provinces since the 1980s [20]. In Chinese Mainland, evidence on human yersiniosis remains limited aside from two outbreaks reported in the 1980s; yersiniosis is not nationally notifiable, and routine clinical testing is uncommon [21,22]. These articles comprised point-prevalence surveys with limited geographic coverage and non-standardization techniques, leading to a lack of systematic epidemiological overview. In addition, these marked inconsistencies in prevalence, whether linked to animal species, sample type, or region, highlight the urgent need for a standardized, nationwide risk assessment framework [23]. However, in the context of China, specific challenges persist regarding the control of *Y. enterocolitica*. Food safety practices vary significantly across the country, particularly concerning slaughterhouse hygiene and cold-chain logistics, which are critical for controlling psychrotrophic pathogens [24]. Furthermore, substantial surveillance gaps exist; unlike *Salmonella* sp. or *Escherichia coli*, *Y. enterocolitica* is not always included in routine national monitoring programs [25]. Additionally, regional variations in livestock practices—ranging from intensive industrial farming in Eastern and Southern regions to traditional free-range herding in Western and Northern areas—create complex epidemiological patterns that complicate uniform control measures [26]. To fill this knowledge gap, a meta-analysis was conducted to determine the prevalence of *Y. enterocolitica* in China from 2000 to 2024. We further assessed potential risk-factors, including geographical location (region, province, longitude, latitude, altitude), animal species, detection methods, study year, and climate variables (annual average temperature, rainfall, humidity) associated with prevalence.

## 2. Methods

### 2.1. Search Strategy

An extensive literature search was carried out following PRISMA (Preferred Reporting Items for Systematic Reviews and Meta-Analyses) guidelines to identify studies reporting the prevalence of *Y. enterocolitica* in economic animals, including cattle, pigs, sheep, and goats in Chinese Mainland [27]. Briefly, studies published between 1 January 2000 and 1 January 2025 were considered in order to capture long-term temporal trends in the prevalence of *Y. enterocolitica* in Chinese livestock and related products. The literature searches were conducted separately in English-language databases (PubMed and ScienceDirect) and Chinese-language databases (CNKI, Wanfang, and VIP), using equivalent search terms translated into English and Chinese.

All records retrieved from the different databases were imported into EndNote (version X.21). Duplicates were first identified and removed automatically based on title, author, publication year, and journal information, followed by manual verification to ensure accuracy. When the same study was identified in both English and Chinese databases, a single record was retained for screening, with preference given to the most recent and complete version of the article.

### 2.2. Search Terms

For PubMed, the search used a combination of MeSH terms and entry terms related to the pathogen (*Y. enterocolitica*), relevant animal hosts, and geographic location. The search strategy was:

((“*Y. enterocolitica*”[Mesh]) OR (“Bacterium enterocoliticum”)) AND ((“Cattle”[Mesh]) OR (Cow) OR (Yak) OR (“Goats”[Mesh]) OR (Ovis aries) OR (Capra) OR (“Swine”[Mesh]) OR (Pig)) AND ((“China”[Mesh]) OR (People’s Republic of China) OR (Mainland China) OR (Inner Mongolia) OR (Manchuria) OR (Sinkiang)).

In ScienceDirect, a keyword-based search was employed using the terms:

“*Y. enterocolitica*” AND “China”, with results restricted to include only research articles.

For the Chinese databases, including the CNKI, Wanfang, and VIP, searches were conducted using the Chinese keywords:

“Xiao Chang Jie Chang Yan Ye Er Sen” AND (Niu OR Yang OR Zhu). To ensure sensitivity, fuzzy search and synonym expansion were applied where available, incorporating additional terms such as “Ye Er Sen Bing”, and “Xiao Chang Jie Chang Yan Ye Er Sen Shi Jun”.

### 2.3. Selection Criteria

Eligible studies were selected based on the following criteria:

#### 2.3.1. Inclusion Criteria

❖ Studies focused on *Y. enterocolitica* in livestock (cattle, pigs, sheep, and goats) in the Chinese Mainland;
❖ Published between 2000 and 1 August 2025;
❖ Studies must report both the total sample size and the *Y. enterocolitica* prevalence;
❖ Studies must include an adequate sample size, ≥30 animals.

#### 2.3.2. Exclusion Criteria

❖ Duplicate data (i.e., use of a dataset from another included study);
❖ Unavailability of the full text;
❖ Sample size was <30;
❖ Data was incomplete or internally contradictory;
❖ The study was conducted outside of Chinese Mainland;
❖ The prevalence of *Y. enterocolitica* was not reported;
❖ Sampling occurred before the year 2000.

### 2.4. Data Extraction

Five reviewers (RZ, SS, SRQ, HYL, and ZZQ) independently extracted the data, which were then compiled into a Microsoft Excel 2019 (version 16.0) database [28]. To ensure accuracy, any discrepancies or uncertainties in the extracted data or study eligibility were resolved through consensus among the authors of this review. Extracted variables included: bibliographic information (first author, year of publication), sampling data (year, season, region), methodological parameters (detection method, sample type), and outcome data (total samples, positive cases). Furthermore, relevant geographic and meteorological data were compiled. Geographic coordinates (longitude, latitude, and altitude) were recorded for each sampling site. Rainfall, temperature, and humidity data for each study period were obtained from the China Meteorological Data Service Center.

### 2.5. Quality Assessment

Quality scoring was performed on the Grading of Recommendations Assessment, Development, and Evaluation methods (GRADE) [29], each study was evaluated using a standardized set of criteria and assigned a quality score ranging from 1 to 4. Each study received 1 point for meeting the following quality criteria:

❖ Usage of a random sampling method;
❖ Well-defined detection assay;
❖ Adequate information on sample collection;
❖ Detailed description of sampling procedures;
❖ Analysis of four or more potential risk factors;

Studies with a score of 4 met all quality criteria and were considered to have a low risk of methodological bias. Studies scoring 1–3 exhibited some methodological limitations, such as incomplete reporting of sampling procedures or a limited assessment of potential risk factors. However, these limitations were not considered sufficient to affect the reliability of prevalence estimates. Therefore, studies with scores of 1–3 were included in the meta-analysis together with those scoring 4. Studies with a score of 0 would have been excluded because they contained too few potential risk factors and did not meet the predefined quality rating criteria. Notably, none of the studies included in this meta-analysis received a score of zero.

### 2.6. Statistical Analysis

Meta-analysis was performed according to PRISMA guidelines (Supplementary Table S1). All analyses were performed in R version 4.4.1 using the “meta” package (version 8.0.1) [27,30]. Before conducting the meta-analysis, we first assessed the normality of the data. Normality tests were applied to both the original prevalence rates and their transformed values using five common transformation methods: original rate (PRAW), logit transformation (PLOGIT), logarithmic conversion (PLN), arcsine transformation (PAS), and double-arcsine transformation (PFT) (Table 1).

The objective was to identify the transformation that most closely approximated a normal distribution. Based on the test outcomes, the Freeman–Turkey double arcsine transformation was selected for conversion. The PFT was applied for both the combined analysis and the separate analysis of cattle, sheep, and goats. However, for the separate analysis of pigs, the PLN was used (Supplementary Table S2). Heterogeneity among studies was assessed using Cochran’s Q-test and *I*^2^ statistics. Potential bias was examined through funnel plots and further assessed by Egger’s test and the Trim-and-Fill method. Sensitivity analysis was also conducted. After that, we performed a subgroup analysis and meta-regression analysis to further investigate heterogeneity sources. The subgroup analysis was based on region, study period, sample classification, detection methods, species, and quality points. To further investigate heterogeneity, we performed subgroup analysis stratified by geographical factors and evaluated longitude, latitude, altitude, rainfall, average annual temperature, humidity, and climate. Details of the R code applied in the meta-analysis are available in (Supplementary Table S3). Additionally, our meta-analysis was not formally registered and did not include a review agreement such as Cochrane registration.

## 3. Results

### 3.1. Search Results and Eligible Studies

**Figure 1:**
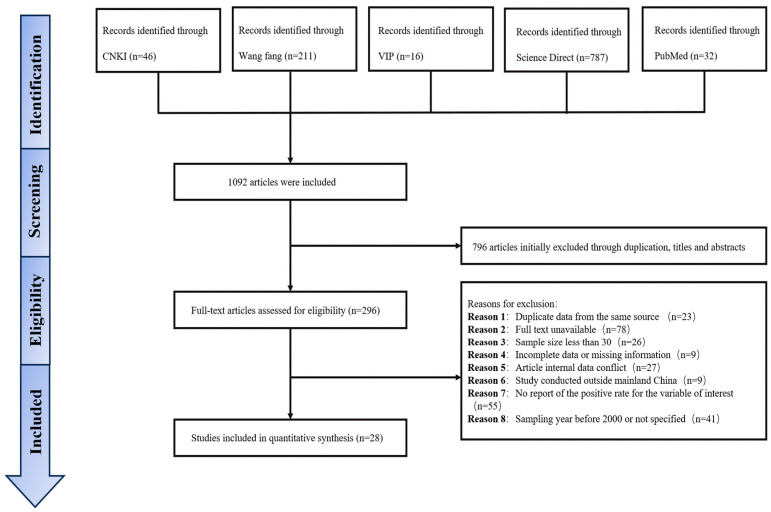
The flow diagram illustrates study selection according to the established eligibility criteria.

From six databases,1092 articles were retrieved, and 28 were qualified for inclusion in the combined meta-analysis (Figure 1, Supplementary Table S4).

Included studies and quality scores for pigs, cattle, sheep, and goats are shown in (Supplementary Tables S4–S7). Quality assessment showed that only 3 articles were assigned scores of 1–2, and the remaining 25 articles were assigned scores of 3–4 (Table 2).

### 3.2. Publication Bias and Sensitivity Analysis

**Figure 2:**
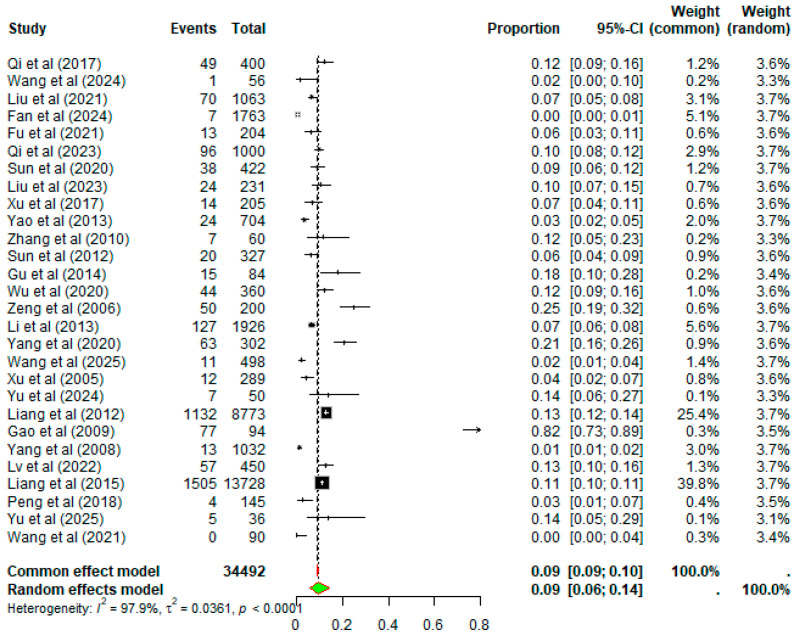
Forest plot of random-effects models shows the pooled prevalence (%) of *Y. enterocolitica* in China with 95% confidence intervals (CI). Individual studies are identified by first author and year, with full reference details provided in the reference list. Between-study heterogeneity was quantified using the *I*^2^ statistic, and statistical significance was assessed using Cochran’s Q test (*p*-value).

Due to high heterogeneity (*I*^2^ = 97.9% and *p* < 0.0001), a random effects-model was applied in the meta-analysis (Figure 2).

**Figure 3:**
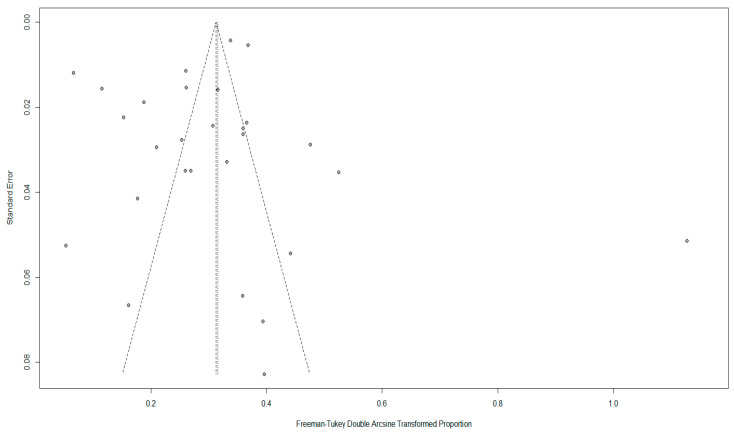
Funnel plot with pseudo 95% confidence interval limits used to assess publication bias among studies reporting *Y. enterocolitica* prevalence; statistical asymmetry was evaluated using standard funnel plot interpretation.

**Figure 4:**
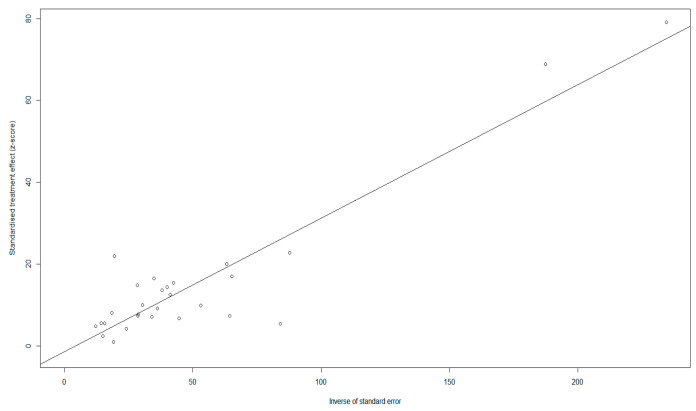
Egger’s test for publication bias.

**Figure 5:**
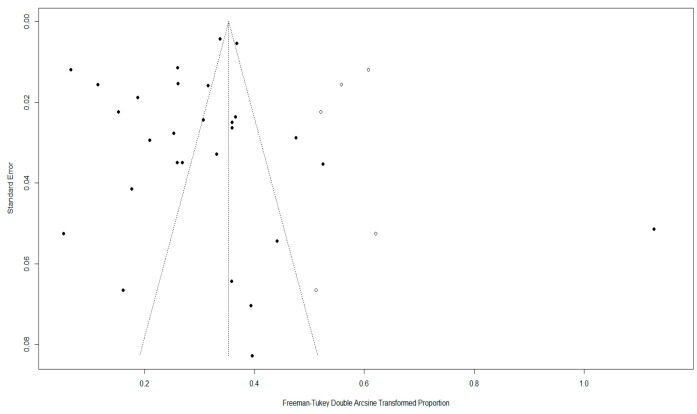
Trim-and-fill method for publication bias.

Although there was asymmetry in the funnel plot (Figure 3), Egger’s test showed no publication bias (*p* > 0.05) (Figure 4), indicating the existence of small-study effects rather than actual bias (Supplementary Table S8). Five missing studies were found using trim-and-fill analysis, but their inclusion had little effect on the pooled prevalence, confirming the stability of our results (Figure 5).

The meta-analysis results and publication bias assessments for each subgroup in the combined analysis are presented in (Supplementary Figures S1–S12). Sensitivity analysis indicated that removing any single study did not substantially affect the pooled prevalence; thus, these results confirm the robustness and reliability of the meta-analysis (Figure 6).

### 3.3. Meta-Analysis of Y. enterocolitica in the Chinese Mainland

The meta-analysis, comprising 28 studies with a total of 34,492 samples, revealed that a pooled prevalence of *Y. enterocolitica* in pigs, cattle, sheep, and goats in China was 9.37% (95% CI: 5.55–14.03) since 2000 (Table 3 and Table S9). The prevalence of *Y. enterocolitica* detected in 2015 or earlier was the highest, 9.69% (95% CI: 4.78–16.04), while the prevalence declined to 3.48% (95% CI: 0.42–8.77) in later years (*p* < 0.05). Prevalence varied across different regions in China (Table 3). Within the regional subgroup analysis, the highest prevalence was observed in the Southern region, 25.00% (95% CI: 19.23–31.25), while the Southwestern region showed the lowest prevalence, 1.78% (95% CI: 0.36–4.20), with *p* < 0.05. Provincially, the highest prevalence was observed in Heilongjiang, 50.88% (95% CI: 44.36–57.40), while the lowest prevalence was in Liaoning, 2.21% (95% CI: 1.08–3.71) (Table 4).

The analysis of various detection methods revealed that the highest prevalence rate was obtained with loop-mediated isothermal amplification, 81.91% (95% CI: 73.42–89.11) and qPCR 10.79% (95% CI: 7.34–14.78) while the lowest prevalence was detected by culture-based 4.60% (95% CI: 2.47–7.30), with (*p* < 0.05). In terms of species, pigs were the most affected, with the highest prevalence of 9.93% (95%: CI 5.79–14.97) compared to cattle 4.67% (95% CI: 1.88–8.47), with (*p* < 0.05). The type of sample substantially influenced detection rates. Prevalence was highest in meat samples at 15.47% (95% CI: 1.99–37.54) in contrast to stool samples 7.23% (95% CI: 4.74–10.19), with (*p* > 0.05). With respect to study quality, studies with lower quality scores (1–2) reported a higher prevalence of 28.51% (95% CI: 0–82.49) compared with those of higher quality (3–4), 7.75% (95% CI: 5.41–10.47), with (*p* < 0.05) (Table 3 and Table S9).

The subgroup analysis for pigs produced results that were consistent with the overall combined analysis (Table 5 and Table S10).

In a separate analysis of cattle, the highest prevalence rate was observed in the Northwestern region at 13.44% (95% CI: 2.30–31.40), and studies conducted between 2016 and 2020 reported a comparatively higher prevalence of 6.27% (95% CI: 0.82–15.57) (Table 6 and Table S11).

For sheep and goats, the highest prevalence occurred in the Eastern region at 3.13% (95% CI: 0.51–7.42), while studies published in 2021 or later showed the highest temporal prevalence at 5.74% (95% CI: 2.19–10.67) with (*p* > 0.05) (Table 7 and Table S12).

In the separate subgroup analyses for cattle, sheep, and goats, both meat samples and PCR-based detection showed the highest prevalence (Table 6, Table 7, Tables S11 and S12).

Geographical factors were also analyzed and found that the regions with a latitude of 40–50° had the highest prevalence, 15.13% (95% CI: 0.00–50.41), compared to regions with 30–40° latitude, 8.70% (95% CI: 5.86–12.02), with (*p* > 0.05). The regions having 113–117° longitude had the highest prevalence, 14.66% (95% CI: 8.17–22.61), compared to regions with ≤112° longitude, 5.35% (95% CI: 3.05–8.22), with (*p* < 0.05). The regions having low altitude <1000 m had the highest prevalence 12.02% (95% CI: 6.80–18.44) compared to regions with >10,000 high altitude 5.99% (95% CI: 3.88–8.50), with (*p* > 0.05). Based on the temperature analysis, regions with an average temperature > 17 °C showed the highest prevalence, 17.65% (95% CI: 5.82–33.93), in contrast to regions with a 15–17 °C temperature, 6.28% (95% CI: 3.55–9.69), with (*p* < 0.05). Regions with rainfall levels ≥ 120 mm exhibited a higher prevalence 12.23% (95% CI: 4.29–23.30), while areas with rainfall between 60 and 119.9 mm showed a lower prevalence 8.61% (95% CI: 3.53–15.57), with (*p* > 0.05). Regions with moderate humidity, 40–55%, had the highest prevalence, 12.33% (95% CI: 4.43–23.25), relative to regions having high humidity, 70–85%, which showed 7.34% (95% CI: 3.53–12.34), with (*p* > 0.05). Temperate monsoon climate regions had the highest prevalence at 11.69% (95% CI: 5.90–19.06) compared to regions of plateau and mountain climate 7.65% (95% CI: 4.87–10.96), with (*p* < 0.05) (Table 8 and Table S13).

The univariate meta-regression showed that region, study period, detection methods, species, quality points, longitude, and average annual temperature may be sources of heterogeneity (*p* < 0.05).

## 4. Discussion

The pooled prevalence of *Y. enterocolitica* in livestock across Chinese Mainland was 9.37% (95% CI: 5.55–14.03), indicating that the pathogen remains a significant public health concern [59]. This finding is particularly relevant in the context of China’s rapidly evolving meat sector, where pork continues to be the dominant animal protein and demand for beef and mutton has increased in recent years [60,61]. Consistent with earlier nationwide data, *Y. enterocolitica* has been detected in multiple livestock species—including pigs, cattle, and sheep—across diverse regions of China [62]. At the food level, a multi-city survey reported the presence of *Y. enterocolitica* in retail food samples, with detections most frequently observed in raw meat products [63]. Internationally, the reported prevalence of *Y. enterocolitica* in livestock and meat products varies widely across countries, likely due to differences in livestock production systems, slaughter hygiene, and surveillance or detection capacity [64,65]. In China, national food-safety risk monitoring and foodborne-disease surveillance networks have expanded and standardized reporting practices, thereby strengthening routine detection and outbreak documentation [66,67]. Notably, our study observed a significant decline in prevalence from 9.69% before 2015 to 3.48% in years thereafter. This downward trend coincides with the broad restructuring of China’s animal agriculture sector after 2015, including the shift toward large-scale standardized farming and the modernization of cold-chain logistics. Together, these changes have contributed to reduced contamination risks along the meat production chain [68,69].

The prevalence of *Y. enterocolitica* varied markedly among host species, with pigs showing the highest prevalence (9.93%), followed by cattle (4.67%), and sheep/goats (1.44%) (Table 3 and Table S9). The disproportionately high prevalence in pigs is consistent with extensive experimental evidence demonstrating that porcine intestinal physiology provides particularly optimal conditions for *Y. enterocolitica* colonization [70,71]. The relatively neutral pH of the porcine cecum and colon (typically 6.0–6.4 in the cecum and 6.1–6.6 in the colon), compared with the more acidic bovine rumen (often ~5.8–6.5 in grain-fed cattle and sometimes falling below ~5.6 during acidosis), may favor bacterial survival and replication [72,73]. This aligns with the global consensus that pigs constitute the major natural reservoir for *Y. enterocolitica* that infects humans, particularly bio-serotype 4/O:3 and other pathogenic lineages commonly implicated in human yersiniosis [74]. These bio-serotypes typically colonize the tonsils and intestinal tract asymptomatically and are shed at high rates, contributing to persistent contamination in transport vehicles, slaughterhouses, and farm environments [75,76]. In contrast, small ruminants (sheep and goats, analyzed together due to limited individual sample sizes) showed the lowest prevalence (2.84%). This reduced susceptibility may partly reflect ruminant digestive physiology: ruminal fermentation produces substantial amounts of short-chain or volatile fatty acids that lower ruminal pH, and these organic acids can markedly reduce *Y. enterocolitica* survival under acidic conditions [77]. It is noteworthy that isolates from cattle and sheep frequently belong to Biotype 1A, which has traditionally been considered less virulent, although its pathogenic potential remains debated when compared with the swine-associated bio-serotype 4/O:3 [78,79]. Nonetheless, the presence of this pathogen in herbivorous livestock should not be overlooked. Biotype 1A strains are increasingly recognized as carriers of virulence-associated markers and clinically relevant antimicrobial resistance phenotypes, suggesting that they may serve as a reservoir of genetic and resistance determinants in farm-associated ecosystems [80,81]. Furthermore, the detection of *Y. enterocolitica* in grazing animals often indicates fecal contamination of pastures, water sources, or silage, serving as a warning signal of compromised biosecurity in mixed-farming systems [82,83]. Similar host patterns have been reported in Europe, where slaughter pigs represent the primary reservoir, while detection in cattle, sheep, and goats remains sporadic [84]. Furthermore, compared with cattle and small-ruminant production, China’s pig industry is highly intensive and vertically integrated, creating favorable conditions for pathogen transmission and persistence [85,86]. Taken together, these findings highlight the need to prioritize surveillance and control measures within the pig and pork production chain, including hygiene management, transport biosecurity, slaughterhouse sanitation, robust tonsil-focused monitoring, and validated isolation and detection methods [87].

Our meta-analysis revealed a marked regional variation in *Y. enterocolitica* prevalence across China. The highest levels were observed in the Southern, Northeastern, and Northern regions, at approximately 25%, 20.6%, and 15.3%, respectively. At the provincial level, Heilongjiang (50%) and Beijing (38.9%) in the North, along with Guangdong (25%) in the South (Table 4), show comparatively high prevalence. These patterns may reflect differences in production systems, livestock density, and ecological conditions, and were consistent with reports showing that pathogenic *Y. enterocolitica* in China was dominated by serogroups O:3 and O:9 and displays province-level clustering [88,89]. Heilongjiang is not only a major pork-producing province but also a key region for dairy cattle and sheep farming in China [90,91]. Accordingly, the elevated pooled prevalence may reflect the combined influence of intensive production and climatic conditions that shape environmental persistence and exposure opportunities. Beijing’s elevated prevalence (38.90%) warrants cautious interpretation. As the nation’s capital, Beijing is covered by intensive food-safety surveillance and laboratory-based monitoring programs; therefore, elevated detection rates may partly reflect greater sampling and testing capacity, rather than a uniformly higher underlying burden [92,93]. Moreover, Beijing sources livestock from multiple provinces, meaning the observed prevalence likely reflects a composite of inter-provincial contamination rather than local production characteristics. Additionally, in Southern urban regions (including Guangdong), consumer preferences for “fresh” meat sold in markets increase handling frequency and the number of contact points along the retail chain, potentially elevating contamination and exposure risks [94,95]. This regional distribution is consistent with the biological characteristics of *Y. enterocolitica*, a psychrotrophic pathogen capable of surviving and proliferating at low temperatures (e.g., 4 °C). The cold climate in Northern regions likely prolongs the pathogen’s environmental persistence and enhances survival during cold-chain transport [96]. Although our meta-regression initially suggested that average temperature and precipitation might be potential risk factors, the extremely high prevalence in the cold Northern provinces points to a more complex interaction. It is plausible that the intensive indoor housing required during long, harsh winters in Northern China plays a decisive role. Overcrowding and poor ventilation in overwintering sheds create a humid, high-density microenvironment that facilitates rapid fecal-oral transmission, potentially amplifying infection rates beyond what ambient climatic factors alone would predict [97].

Climate, production practices, and environmental conditions jointly shape geographic variation in *Y. enterocolitica* occurrence across China [98,99]. Higher prevalence was observed in low-altitude regions (Table 8 and Table S13), which in China often overlap with major agricultural plains where livestock production is concentrated [100]. With economic development and population growth, many regions have transitioned from dispersed smallholder systems to larger-scale and more specialized production, increasing animal density and contact rates and thereby facilitating fecal–oral transmission and indirect spread through contaminated environments [101]. The pathogen was more frequently detected in areas characterized by heavier rainfall, higher humidity, and warmer temperatures (Table 8 and Table S13). Such wet and warm conditions can enhance environmental dissemination of foodborne pathogens (e.g., via runoff, surface water contamination, and wastewater), thereby increasing opportunities for food-chain contamination [102,103]. Temperature-dependent regulation of virulence and thermally modulated biofilm formation further enhances the ability of *Y. enterocolitica* to persist under fluctuating environmental conditions [104,105]. This pattern aligns with the higher prevalence observed in temperate monsoon regions (Table 8 and Table S13), where strong seasonality in rainfall and humidity can elevate contamination pressure across the environment [106,107]. These findings highlight the need to integrate regional meteorological and agricultural characteristics into surveillance and control frameworks to strengthen food safety management [108].

The subgroup analysis revealed distinct differences in prevalence across detection methods, reflecting inherent variation in assay sensitivity, analytical targets, and culture recoverability. In our meta-analysis, only a single study reported LAMP (Loop-mediated isothermal amplification), with a prevalence rate of 81.91%. Because this estimate is based on just one dataset, it lacks the precision and external validity of the eight independent studies contributing to the qPCR pooled estimate. Moreover, the LAMP study used a simple processing protocol that differed from the standardized quantitative thresholds applied in qPCR, further limiting comparability. Therefore, qPCR yielded the highest prevalence (10.79%) (Table 3 and Table S9), whereas culture-based methods produced the lowest (Table 3 and Table S9), consistent with international evidence that molecular assays generally detect two to three times more positives than traditional culture [109]. Lower prevalence in culture-based approaches is expected, as *Y. enterocolitica* grows slowly, competes poorly with background flora, and readily enters a viable but non-culturable (VBNC) state under refrigeration or environmental stress, making routine isolation difficult [110,111]. These hard-to-recover cells and low-level contaminations often yield false negatives under the standard EN ISO 10273:2017 workflow, especially in samples with abundant competing flora [10,110]. In contrast, molecular methods such as qPCR and PCR directly detect DNA without requiring viable bacteria, allowing detection of both viable cells and non-viable remnants. This may inflate apparent positivity compared with culture-based methods [112]. qPCR is particularly sensitive, and can detect low-copy-number targets (e.g., ail, ystA, and inv) even when bacterial loads are minimal [112]. However, because qPCR cannot distinguish between viable and non-viable organisms, it may overestimate infection risk. The PFGE remains a reliable, high-resolution subtyping method for outbreak investigations and assessing clonal relatedness [113], but its performance depends on laboratory conditions and electrophoretic parameters. Importantly, PFGE cannot directly detect *Y. enterocolitica*; it requires prior successful culture isolation. Thus, the moderate prevalence observed in PFGE-based studies reflects limitations of primary culture—such as slow growth, microbial competition, and VBNC states—rather than the intrinsic sensitivity of PFGE [114,115]. A more unified detection framework—using molecular assays for rapid screening, culture as the confirmatory gold standard, and incorporating standardized cold-enrichment with multi-target detection—would improve sensitivity, reduce false negatives, and enhance the reliability and comparability of surveillance data [116]. Therefore, the observed decline in prevalence over time (Table 3 and Table S9) should be interpreted with caution, as shifts in laboratory methodology (from culture to PCR/CIDTs) in recent years may partially confound temporal trends in surveillance data [117,118]. Significant variation in prevalence was observed across sample types, reflecting the organism’s tissue tropism, contamination routes during slaughter, and differences in detectability among biological matrices [119]. Notably, the prevalence of meat and meat products (15.47%) was more than double that of fecal samples (7.23%). This discrepancy is largely driven by the swine sector, where tonsillar carriage is common and can seed carcass contamination during slaughter [120]. This seemingly counterintuitive finding indicates that cross-contamination during slaughter and processing acts as a major amplification step [120]. Contamination frequently occurs during scalding/dehairing and evisceration, and may be further spread during carcass splitting, when intestinal contents spill onto the carcass surface; additionally, tonsillar and oropharyngeal sources contribute substantially to cross-contamination [121,122]. Poor hygiene, inadequate tool disinfection, and cross-contamination via knives, workers’ hands, and other contact surfaces further elevate contamination levels [123,124]. Several studies report that contamination in raw pork can reach up to 30% in some settings, although estimates vary by country, sampling design, and detection method [125]. In ruminants, contamination dynamics differ because dehiding, rather than scalding/dehairing, is the dominant source of carcass contamination. During cattle and sheep processing, hide removal can transfer surface contaminants to the underlying carcass if not properly controlled [121]. Consequently, beef and mutton generally show lower detection rates of pathogenic *Y. enterocolitica* compared with pork; in small ruminants, detections are dominated by non-pathogenic lineages, with pathogenic strains identified only sporadically [126,127]. Accordingly, contamination in ruminant meat is more plausibly linked to hide/pelt–to–carcass transfer during dressing and evisceration, whereas pigs typically harbor pathogenic strains in the tonsils and tongue, which often show higher positivity than fecal or rectal samples [128,129]. Unlike pigs, which undergo scalding and dehairing with the skin intact, cattle and sheep carcasses are dressed by complete dehiding, increasing the risk of contamination transfer if hygiene lapses occur [128]. Due to its psychrotrophic nature, *Y. enterocolitica* can survive and even multiply at refrigeration temperatures (4–7 °C) [130]. This cold tolerance allows the pathogen to persist during chilled storage and throughout the retail cold chain, meaning that detection in chilled meat may remain substantial even when fecal or rectal samples show lower positivity [130,131]. Similar findings have been reported in slaughterhouses in Finland, Denmark, and Switzerland, where meat and oral samples consistently showed higher prevalence, largely influenced by sanitation practices and the degree of automation along the slaughter line [127,131]. In contrast, fecal samples may yield a lower apparent positivity because fecal matrices often contain PCR inhibitors and require matrix-specific nucleic-acid extraction; inadequate preparation can decrease sensitivity and lead to false negatives [132]. Given these differences, control strategies should prioritize the pig–pork chain by optimizing pre-slaughter feed withdrawal/fasting and implementing targeted interventions for tonsil-and head-associated contamination sources [131,133]. Additionally, validated decontamination strategies (e.g., organic-acid sprays) when integrated with good manufacturing practices can further reduce *Y. enterocolitica* loads on pork products [134].

While this study provides a comprehensive overview, several limitations should be interpreted with caution. Firstly, despite exhaustive efforts to retrieve all the eligible literature from major databases, the geographic representation of included studies remains uneven. Most data originated from Eastern and Northern China, whereas vast regions in Western China (e.g., Tibet, Xinjiang) remain under-surveilled. Nonetheless, because Eastern and Northern China constitute the country’s primary livestock production and consumption hubs, the current dataset still offers the most informative baseline available for national policy formulation, even though the epidemiological landscape in the West requires further investigation. Secondly, inconsistencies in sampling methods across primary studies limited our ability to make more granular comparisons. Many studies did not differentiate between backyard and intensive farming systems, preventing direct meta-regression analysis of how production type influences infection risk. Substantial heterogeneity was observed across diagnostic methods. Variations in detection sensitivity—ranging from traditional culture to high-sensitivity qPCR—introduced unavoidable heterogeneity into the pooled estimates. While molecular assays generally provide higher sensitivity, culture-based methods confirm the presence of viable organisms. To account for these differences, the detection method was treated as a distinct variable in subgroup analyses. Finally, a potential publication bias cannot be excluded, as studies reporting negative results are less likely to be published, potentially leading to an overestimation of prevalence. However, the overall outcomes of our study remain relatively robust and likely reflect the broader prevalence of *Y. enterocolitica* among domestic animals in the Chinese Mainland.

This meta-analysis was not prospectively registered. Although this may reduce transparency compared with registered reviews, predefined eligibility criteria were applied throughout the study to minimize bias. Furthermore, the included studies were predominantly cross-sectional, which limits the ability to infer causal relationships. Other limitations include geographic concentration of available studies, heterogeneity in sampling approaches, and potential publication bias.

## 5. Conclusions

This meta-analysis indicates that *Y. enterocolitica* remains widely distributed throughout China, with clear host-specific and regional differences influenced by production practices, agricultural intensity, and climatic conditions. The consistently high prevalence in pigs confirms their role as the primary reservoir of pathogenic bioserotypes. While an overall decline in prevalence has occurred over time, persistently high levels in certain regions, particularly South China, continue to raise public health and food safety concerns. Considerable heterogeneity was observed across regions, host species, sample types, and diagnostic methods, with geographic and climatic factors contributing to the observed variability. From a One Health perspective, these findings highlight the need for strengthened, region-specific surveillance and control measures, especially in high-prevalence areas, with explicit consideration of differences between intensive and extensive production systems, as variations in animal density, management practices, and environmental exposure may influence transmission dynamics. In addition, the use of standardized and sensitive diagnostic methods is essential to improve comparability across regions and to support targeted control efforts across the food production chain.

## Figures and Tables

**Figure 6:**
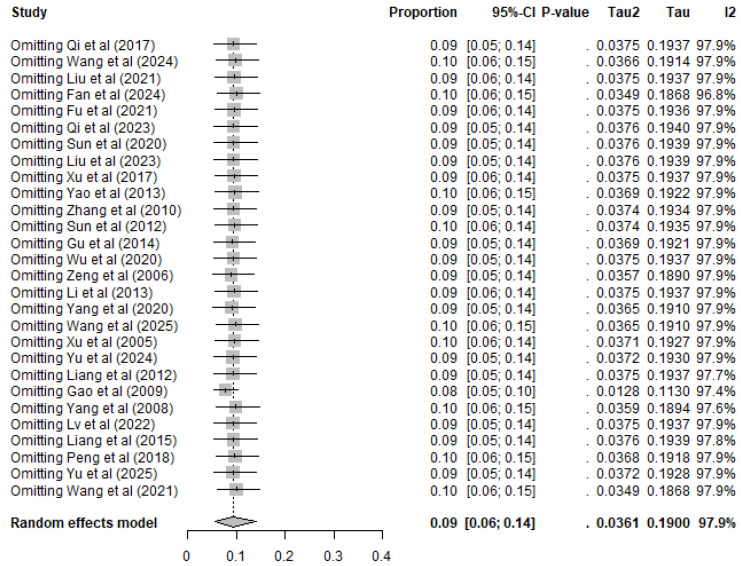
Sensitivity analysis. Individual studies are identified by first author and year, with full reference details provided in the reference list.

**Table 1:** Normality tests for original and transformed rate distributions (pigs + cattle + sheep and goats).

Conversion Form	W	P
PRAW	0.56065	5.11 × 10^−8^
PLN	NaN	NA
PLOGIT	NaN	NA
PAS	0.78766	6.518 × 10^−5^
PFT	0.7885	6.738 × 10^−5^

PRAW: original rate, PLN: logarithmic conversion, PLOGIT: logit transformation, PAS: arcsine transformation, PFT: double-arcsine transformation, NaN: meaningless number, NA: missing data.

**Table 2:** Selected studies of *Y. enterocolitica* in the Chinese Mainland.

References	Sampling Time	Detection Method *	No. Tested	No. Positive	Prevalence (%)	Quality Score
**Northwestern**						
Qi et al. (2017) [31]	2017	PCR	400	49	12.25	4
Xu et al. (2017) [32]	UN	Culture-based	205	14	6.83	3
Zhang et al. (2010) [33]	2009	PCR	60	7	11.7	4
Lv et al. (2022) [34]	2018–2019	PCR	450	57	12.67	4
Liang et al. (2012) [35]	2009–2011	PCR	1930	98	5.08	4
**Northeastern**						
Wang et al. (2025) [36]	2018–2021	PCR	498	11	2.21	4
Liang et al. (2012) [35]	2009–2011	PCR	226	115	50.88	4
**Northern**						
Li et al. (2013) [37]	2001–2010	Culture-based	1926	127	6.59	4
Liang et al. (2012) [35]	2009–2011	PCR	2531	288	15.33	4
**Southern**						
Zeng et al. (2006) [38]	M	Culture-based	200	50	25	3
**Southwestern**						
Fan et al. (2024) [39]	2017–2022	Culture-based	1763	7	0.4	4
Yao et al. (2013) [40]	UN	Culture-based	704	24	3.41	3
Liang et al. (2012) [35]	2009–2011	PCR	2042	49	2.40	4
**Central**						
Li et al. (2013) [37]	2001–2010	Culture-based	1926	127	6.59	4
Liang et al. (2012) [35]	2009–2011	PCR	1582	370	23.39	4
**Eastern**						
Wang et al. (2024) [41]	2022	Culture-based	56	1	1.79	4
Liu et al. (2021) [42]	2018–2019	Culture-based	1063	70	6.59	4
Fu et al. (2021) [43]	2019–2020	Culture-based	204	13	6.37	4
Qi et al. (2023) [44]	UN	Culture-based	1000	96	9.6	3
Sun et al. (2020) [45]	2018	Culture-based	422	38	9	4
Liu et al. (2023) [46]	2021	qPCR	231	24	10.39	4
Sun et al. (2012) [47]	2010	PFGE	327	20	6.12	4
Gu et al. (2014) [48]	2012	Culture-based	84	15	17.86	4
Wu et al. (2020) [49]	2018.08–2019.07	Culture-based	360	44	12.22	4
Yang et al. (2020) [50]	2011–2013	Culture-based	302	63	20.86	4
Xu et al. (2005) [51]	1-10-2024	PCR	289	12	4.15	4
Yu et al. (2024) [52]	2022–2023	PCR	50	7	14	4
Yang et al. (2008) [53]	2004–2006	Culture-based	1032	13	1.26	4
Yu et al. (2025) [54]	UN	M	36	5	13.89	2
Liang et al. (2012) [35]	2009–2011	PCR	462	112	24.24	4
**M**						
Gao et al. (2009) [55]	UN	LAMP	94	77	81.91	2
Liang et al. (2015) [56]	UN	PFGE	13728	1505	10.96	3
Peng et al. (2018) [57]	UN	M	145	4	2.76	1
Wang et al. (2021) [58]	2015–2016.	Culture-based	90	0	0	4

Detection methods *: PCR: Polymerase Chain Reaction; qPCR = quantitative polymerase chain reaction; PFGE: Pulsed-field gel electrophoresis; LAMP: Loop-mediated isothermal amplification; UN: Unclear; M: Missing.

**Table 3:** Pooled prevalence of *Y. enterocolitica* in pigs, cattle, sheep, and goats in the Chinese Mainland.

		No. Studies	No. Tested	No. Positive	% (95% CI*)
**Region ^a^**					
	Eastern	15	5918	533	9.44% (6.23–13.20)
	Northeastern	2	724	126	20.64% (0.00–79.67)
	Northern	1	2531	388	15.33% (13.95–16.76)
	Central	2	3508	497	13.91% (1.99–33.98)
	Northwestern	5	3045	225	9.13% (5.86–13.00)
	Southern	1	200	50	25.00% (19.23–31.25)
	Southwestern	3	4509	80	1.78% (0.36–4.20)
**Study period**					
	≤2015	7	12,504	1377	9.69% (4.78–16.04)
	2016–2020	9	4503	285	3.74% (1.32–7.23)
	≥2021	5	1373	48	3.48% (0.42–8.77)
**Sample classification**					
	Intestinal contents	3	9273	1183	8.68% (2.84–17.17)
	Meat	10	3574	233	15.47% (1.99–37.54)
	Oral contents	4	890	96	12.65% (2.87–27.38)
	Stool	14	20,665	1973	7.23% (4.74–10.19)
	Milk	1	90	0.00	0.00% (0.00–1.90)
**Detection methods ^b^**					
	Culture-based	12	8825	447	4.60% (2.47–7.30)
	Pulse-field gel electrophoresis	2	14,055	1525	8.64% (4.56–13.85)
	Loop-mediated isothermal amplification	1	94	77	81.91% (73.42–89.11)
	PCR	6	10,470	1268	8.56% (4.62–13.52)
	qPCR	2	281	31	10.79% (7.34–14.78)
**Species**					
	Cattle	16	2884	172	4.67% (1.88–8.47)
	Pig	26	28,349	3239	9.93% (5.79–14.97)
	Sheep, and goat	10	3259	74	1.44% (0.01–4.29)
**Quality Points**					
	1–2	3	275	86	28.51% (0–82.49)
	3–4	25	34,217	3399	7.75% (5.41–10.47)
**Total**		28	34,492	3485	9.37% (5.55–14.03)

CI*: Confidence interval. Region ^a^: Eastern: Fujian, Shanghai, Jiangsu, Jiangxi, Shandong; Northeastern: Liaoning, Heilongjiang; Northern: Beijing, Inner Mongolia, Tianjin; Northwestern: Ningxia, Qinghai, Shaanxi; Southern: Guangdong; Southwestern: Sichuan, Yunnan; Central: Henan. This classification aligns with the standard geographical divisions defined by the National Bureau of Statistics of China and is widely applied in epidemiological surveillance reports, such as *China CDC Weekly*. Detection Methods ^b^: PCR: Polymerase Chain Reaction; qPCR: quantitative polymerase chain reaction. Culture-based: Refers to the isolation of pure microbial strains through cultivation, followed by identification using biochemical tests.

**Table 4:** Pooled prevalence of *Y. enterocolitica* across provincial regions in the Chinese Mainland.

Province	No. Studies	Region	No. Tested	No. Positive	Prevalence %	% (95% CI)
Fujian	1	Eastern	204	13	6.37	3.38–10.19
Shanghai	2	Eastern	140	16	9.85	5.31–15.48
Guangdong	1	Southern	200	50	25	19.23–31.25
Liaoning	1	Northeastern	498	11	2.21	1.08–3.71
Beijing	1	Northern	700	272	38.86	35.28–42.50
Heilongjiang	1	Northeastern	226	115	50.88	44.36–57.40
Henan	2	Central	3508	497	13.91	1.99–33.98
Jiangsu	8	Eastern	4757	353	7.90	4.75–11.74
Jiangxi	2	Eastern	404	119	36.78	8.53–71.29
Inner Mongolia	1	Northern	759	49	6.46	4.81–8.32
Ningxia	1	Northwestern	1530	72	4.71	3.70–5.83
Qinghai	4	Northwestern	1065	96	8.79	5.80–12.29
Sichuan	2	Southwestern	2590	26	11.3	0.01–3.74
Tianjin	1	Northern	1072	67	6.25	4.87–7.78
Yunnan	2	Southwestern	1919	54	2.83	1.98–3.82
Shaanxi	1	Northwestern	450	57	12.67	9.74–15.91
Shandong	3	Eastern	413	32	9.66	4.05–17.10

**Table 5:** Pooled prevalence of *Y. enterocolitica* in pigs in the Chinese Mainland.

		No. Studies	No. Tested	No. Positive	% (95% CI*)
Region ^a^					
	Eastern	14	3528	415	9.89% (7.16–13.66)
	Northeastern	2	546	118	7.19% (0.14–100)
	Northern	1	2531	388	15.33% (13.99–16.80)
	Central	2	2692	457	13.59% (4.66–39.68)
	Northwestern	4	2690	178	8.42% (5.45–13.02)
	Southern	1	200	50	25.00% (19.66–31.78)
	Southwestern	3	4509	80	1.54% (0.43–5.45)
Study period					
	≤2015	7	13,422	1650	8.85% (5.26–14.88)
	2016–2020	8	3174	188	3.61% (1.56–8.36)
	≥2021	4	1126	40	3.70% (0.97–14.13)
Sample classification					
	Intestinal contents	3	9273	1183	12.84% (12.17–13.54)
	Meat	9	2882	143	7.24% (2.58–20.33)
	Oral contents	3	869	92	9.26% (2.71–31.65)
	Stool	13	15,293	1817	8.09% (5.54–11.80)
Detection methods ^b^					
	Culture-based	12	6283	421	6.81% (3.68–12.60)
	Pulse-field gel electrophoresis	2	11,548	1481	9.61% (4.75–19.43)
	Loop-mediated isothermal amplification	1	94	77	81.91% (74.49–90.08)
	PCR	6	9951	1220	7.22% (3.69–14.14)
	qPCR	2	281	31	11.14% (7.99–15.52)
Quality Points					
	1–2	3	275	86	15.35% (2.22–100)
	3–4	22	28,063	3153	7.50% (5.13–10.97)
Total		25	28,338	3239	8.15% (5.44–12.19)

CI*: Confidence interval. Region ^a^: Eastern: Fujian, Shanghai, Jiangsu, Jiangxi, Shandong; Northeastern: Liaoning, Heilongjiang; Northern: Beijing, Inner Mongolia, Tianjin; Northwestern: Ningxia, Qinghai, Shaanxi; Southern: Guangdong; Southwestern: Sichuan, Yunnan; Central: Henan. Detection Methods ^b^: PCR: Polymerase Chain Reaction; qPCR: quantitative polymerase chain reaction.

**Table 6:** Pooled prevalence of *Y. enterocolitica* in cattle in the Chinese Mainland.

		No. Studies	No. Tested	No. Positive	% (95% CI*)
**Region ^a^**					
	Eastern	10	1128	67	4.08% (0.73–9.45)
	Northeastern	1	178	8	4.49% (1.86–8.11)
	Central	1	485	32	6.60% (4.55–8.99)
	Northwestern	2	355	47	13.44% (2.30–31.40)
**Study period**					
	≤2015	5	1017	48	4.28% (0.03–13.38)
	2016–2020	6	111	1	6.27% (0.82–15.57)
	≥2021	2	703	75	0.52% (0.00–4.61)
**Sample classification**					
	Meat	6	636	87	11.92% (5.08–20.94)
	Stool	9	2158	85	2.45% (0.77–4.88)
	Milk	1	90	0	0.00% (0.00–1.90)
**Detection methods ^b^**					
	Culture-based	11	1727	110	4.68% (1.34–9.60)
	Pulse-field gel electrophoresis	2	760	21	2.64% (1.57–3.95)
	PCR	3	397	41	6.19% (0.00–23.11)
**Quality Points**					
	3–4	16	2884	172	4.67% (1.88–8.47)
**Total**		16	2884	172	4.67% (1.88–8.47)

CI*: Confidence interval. Region ^a^: Eastern: Fujian, Shanghai, Jiangsu, Jiangxi, Shandong; Northeastern: Liaoning, Heilongjiang; Northwestern: Ningxia, Qinghai, Shaanxi; Central: Henan. Detection Methods ^b^: PCR: Polymerase Chain Reaction.

**Table 7:** Pooled prevalence of *Y. enterocolitica* in sheep and goats in the Chinese Mainland.

		No. Studies	No. Tested	No. Positive	% (95% CI*)
**Region ^a^**					
	Eastern	7	1248	51	3.13% (0.51–7.42)
	Central	1	331	8	2.42% (0.99–4.39)
**Study period**					
	≤2015	3	785	19	3.44% (0.03–10.78)
	2016–2020	3	272	3	0.77% (0.00–6.21)
	≥2021	1	122	7	5.74% (2.19–10.67)
**Sample classification**					
	Stool	8	3214	71	2.36% (0.49–5.33)
	Meat	1	42	3	7.14% (0.93–17.32)
**Detection methods ^b^**					
	Culture-based	6	1387	44	1.85% (0.12–5.01)
	Pulse-field gel electrophoresis	2	1747	23	4.35% (0.00–20.26)
	PCR	1	122	7	5.74% (2.19–10.67)
**Quality Points**					
	3–4	9	3256	74	2.62% (0.67–5.57)
**Total**		9	3256	74	2.62% (0.67–5.57)

CI*: Confidence interval. Region ^a^: Eastern: Fujian, Shanghai, Jiangsu, Jiangxi, Shandong; Central: Henan. Detection Methods ^b^: PCR: Polymerase Chain Reaction.

**Table 8:** Geographical factors affecting the prevalence of *Y. enterocolitica* in pigs, cattle, sheep, and goats in the Chinese Mainland.

		No. Studies	No. Tested	No. Positive	% (95% CI*)
**Longitude**					
	≤112°	7	8313	354	5.35% (3.05–8.22)
	113–117°	11	8978	1202	14.66% (8.17–22.61)
	≥118°	8	3144	343	11.31% (4.49–20.62)
**Latitude**					
	20–30°	6	3087	280	14.84% (4.78–29.07)
	30–40°	18	15,865	1444	8.70% (5.86–12.02)
	40–50°	2	1483	175	15.13% (0.00–50.41)
**Altitude (m)**					
	<1000	14	7975	901	12.02% (6.80–18.44)
	1000–10,000	7	7187	727	11.64% (4.07–22.26)
	>10,000	5	5273	271	5.99% (3.88–8.50)
**Rainfall (mm)**					
	<60	9	10,545	1097	10.82% (6.33–16.30)
	60–119.9	10	5701	531	8.61% (3.53–15.57)
	≥120	7	4189	271	12.23% (4.29–23.30)
**Humidity**					
	40–55%	4	3402	425	12.33% (4.43–23.25)
	55–70%	12	10,390	1115	11.99% (6.52–18.80)
	70–85%	10	6643	359	7.34% (3.53–12.34)
**Average annual temperature**					
	<15 °C	8	10,135	1256	11.98% (6.45–18.89)
	15–17 °C	12	9076	416	6.28% (3.55–9.69)
	>17 °C	6	1224	227	17.65% (5.82–33.93)
**Climate**					
	Plateau and mountain climate	4	2595	168	7.65% (4.87–10.96)
	Subtropical monsoon climate	12	7893	510	10.10% (5.00–16.68)
	Temperate monsoon climate	10	9947	1221	11.69% (5.90–19.06)

CI*: Confidence interval.

## Data Availability

The data that support the findings of this study are available from the corresponding author upon reasonable request.

## Supplementary Materials

The following supporting information can be downloaded at: https://www.mdpi.com/article/10.3390/ani16030418/s1, Table S1. PRISMA Checklist. Table S2. The normality test for the original rate and various transformations of the rate. Table S3. The code in R for this meta-analysis. Table S4. Included studies and quality scores for pigs, cattle, sheep, and goats. Table S5. Included studies and quality scores for pigs. Table S6. Included studies and quality scores for cattle. Table S7. Included studies and quality scores for sheep and goats. Table S8. Egger’s test for publication bias. Table S9. Pooled prevalence of *Y. enterocolitica* in pigs, cattle, sheep and goats in Chinese Mainland. Table S10. Pooled prevalence of *Y. enterocolitica* in pigs in Chinese Mainland. Table S11. Pooled prevalence of *Y. enterocolitica* in cattle in Chinese Mainland. Table S12. Pooled prevalence of *Y. enterocolitica* in sheep and goats in Chinese Mainland. Table S13. Geographical factors affecting the prevalence of *Y. enterocolitica* in pigs, cattle, sheep and goats in Chinese Mainland. Figure S1. Funnel plot with pseudo 95% confidence limit intervals for the examination of publication bias in the region subgroup of pigs, cattle, sheep, and goats. Figure S2. Funnel plot with pseudo 95% confidence limit intervals for the examination of publication bias in the study period subgroup of pigs, cattle, sheep, and goats. Figure S3. Funnel plot with pseudo 95% confidence limit intervals for the examination of publication bias in the sample classification subgroup of pigs, cattle, sheep, and goats. Figure S4. Funnel plot with pseudo 95% confidence limit intervals for the examination of publication bias in the detection method subgroup of pigs, cattle, sheep, and goats. Figure S5. Funnel plot with pseudo 95% confidence limit intervals for the examination of publication bias in the species subgroup of pigs, cattle, sheep, and goats. Figure S6. Funnel plot with pseudo 95% confidence limit intervals for the examination of publication bias in the quality points subgroup of pigs, cattle, sheep, and goats. Figure S7. Forest plot of the region subgroup of pigs, cattle, sheep, and goats. Figure S8. Forest plot of the study period subgroup of pigs, cattle, sheep, and goats. Figure S9. Forest plot of the sample classification subgroup of pigs, cattle, sheep, and goats. Figure S10. Forest plot of the detection methods subgroup of pigs, cattle, sheep, and goats. Figure S11. Forest plot with the species subgroup of pigs, cattle, sheep, and goats. Figure S12. Forest plot of the quality points subgroup of pigs, cattle, sheep, and goats.

## Abbreviations

The following abbreviations are used in this manuscript:

PRISMA	Preferred Reporting Items for Systematic Reviews and Meta-Analyses
CNKI	China National Knowledge Infrastructure
*Y. enterocolitica*	*Yersinia enterocolitica*
GRADE	Grading of Recommendations Assessment, Development, and Evaluation methods
LAMP	Loop-mediated isothermal amplification
VBNC	Viable but non-culturable
