# Supplementary material for: Prevalence and Geographical Distribution of Foodborne Yersinia enterocolitica in Chinese Livestock and Their Products: A Systematic Review and Meta-Analysis (2000–2024)
Source: Animals (Basel). 2026 Jan 29;16(3):418. doi: 10.3390/ani16030418 (PMC12896466; doi:10.3390/ani16030418)
Supplement: Supplementary file 1 [file animals-16-00418-s001.zip › animals-4094424-supplementary.pdf]

Table S1. PRISMA Checklist item.

Section/topic	#	Checklist item	Reported on page #
**TITLE**			
Title	1	Prevalence of foodborne Yersinia enterocolitica in Chinese livestock and their products: A systematic review and meta-analysis (2000-2024)	1
**ABSTRACT**			
Structured summary	2	Provide a structured summary including, as applicable: background; objectives; data sources; study eligibility criteria, participants, and interventions; study appraisal and synthesis methods; results; limitations; conclusions and implications of key findings; systematic review registration number.	1
**INTRODUCTION**			
Rationale	3	Describe the rationale for the review in the context of what is already known.	2
Objectives	4	Provide an explicit statement of questions being addressed with reference to participants, interventions, comparisons, outcomes, and study design (PICOS).	3
**METHODS**			
Protocol and registration	5	Indicate if a review protocol exists, if and where it can be accessed (e.g., Web address), and, if available, provide registration information including registration number.	5
Eligibility criteria	6	Specify study characteristics (e.g., PICOS, length of follow-up) and report characteristics (e.g., years considered, language, publication status) used as criteria for eligibility, giving rationale.	4
Information sources	7	Describe all information sources (e.g., databases with dates of coverage, contact with study authors to identify additional studies) in the search and date last searched.	3

Search	8	Present full electronic search strategy for at least one database, including any limits used, such that it could be repeated.	3
Study selection	9	State the process for selecting studies (i.e., screening, eligibility, included in systematic review, and, if applicable, included in the meta-analysis).	3,4，Fig.1
Data collection process	10	Describe method of data extraction from reports (e.g., piloted forms, independently, in duplicate) and any processes for obtaining and confirming data from investigators.	4
Risk of bias in individual studies	12	Describe methods used for assessing risk of bias of individual studies (including specification of whether this was done at the study or outcome level), and how this information is to be used in any data synthesis.	5，Fig.3
Summary measures	13	State the principal summary measures (e.g., risk ratio, difference in means).	4,5
Synthesis of results	14	Describe the methods of handling data and combining results of studies, if done, including measures of consistency (e.g., I^2) for each meta-analysis.	5
Risk of bias across studies	15	Specify any assessment of risk of bias that may affect the cumulative evidence (e.g., publication bias, selective reporting within studies).	5，Fig. 4,5
Additional analyses	16	Describe methods of additional analyses (e.g., sensitivity or subgroup analyses, meta-regression), if done, indicating which were pre-specified.	5
**RESULTS**			
Study selection	17	Give numbers of studies screened, assessed for eligibility, and included in the review, with reasons for exclusions at each stage, ideally with a flow diagram.	5
Study characteristics	18	For each study, present characteristics for which data were extracted (e.g., study size, PICOS, follow-up period) and provide the citations.	6-8
Risk of bias within studies	19	Present data on risk of bias of each study and, if available, any outcome level assessment (see item 12).	11

Results of individual studies	20	For all outcomes considered (benefits or harms), present, for each study: (a) simple summary data for each intervention group (b) effect estimates and confidence intervals, ideally with a forest plot.	9
Synthesis of results	21	Present results of each meta-analysis done, including confidence intervals and measures of consistency.	11-12, 16-17, 18-19，Table 4-8，Table S9-S13
Risk of bias across studies	22	Present results of any assessment of risk of bias across studies (see Item 15).	9
Additional analysis	23	Give results of additional analyses, if done (e.g., sensitivity or subgroup analyses, meta-regression [see Item 16]).	13-20
**DISCUSSION**			
Summary of evidence	24	Summarize the main findings including the strength of evidence for each main outcome; consider their relevance to key groups (e.g., healthcare providers, users, and policy makers).	20-23
Limitations	25	Discuss limitations at study and outcome level (e.g., risk of bias), and at review-level (e.g., incomplete retrieval of identified research, reporting bias).	24
Conclusions	26	Provide a general interpretation of the results in the context of other evidence, and implications for future research.	24
**FUNDING**			
Funding	27	Describe sources of funding for the systematic review and other support (e.g., supply of data); role of founders for the systematic review.	25

From: Moher D, Liberati A, Tetzlaff J, Altman DG, The PRISMA Group (2009). Preferred Reporting Items for Systematic Reviews and Meta-Analyses: The PRISMA Statement. PLoS Med 6(6): e1000097. doi:10.1371/journal.pmed1000097

For more information, visit: **www.prisma-statement.org**.

Table S2. The normality test for original rate and various transformations of the rate.

Pigs

Conversion Form	W	P
PRAW	0.54093	9.717×10^{-8}
PLN	0.91089	0.03191
PLOGIT	0.88972	0.01103
PAS	0.73364	2.139×10^{-5}
PFT	0.73716	2.405×10^{-5}

PRAW: original rate, PLN: logarithmic conversion, PLOGIT: logit transformation, PAS: arcsine transformation, PFT: double-arcsine transformation, NaN: meaningless number, NA: missing data.

Cattle

Conversion Form	W	P
PRAW	0.777	0.00137
PLN	NaN	NA
PLOGIT	NaN	NA
PAS	0.91316	0.1308
PFT	0.91631	0.1471

PRAW: original rate, PLN: logarithmic conversion, PLOGIT: logit transformation, PAS: arcsine transformation, PFT: double-arcsine transformation, NaN: meaningless number, NA: missing data.

Sheep and goat

Conversion Form	W	P
PRAW	0.87804	0.1497
PLN	NaN	NA
PLOGIT	NaN	NA
PAS	0.92476	0.4331
PFT	0.92677	0.4512

PRAW: original rate, PLN: logarithmic conversion, PLOGIT: logit transformation, PAS: arcsine transformation, PFT: double-arcsine transformation, NaN: meaningless number,

NA: missing data.

Table S3. The code in R for this meta-analysis.

Logarithmic conversion (PLN)	rate<-transform [m1, log=log(event/n)]; shapiro.test(rate$log)
Logit transformation (PLOGIT)	rate<-transform{m1, logit=log[(event/n)/(1-event/n)]}; shapiro.test(rate$logit)
Arcsine transformation (PAS)	rate<-transform{m1, arcsin.size=asin[sqrt(event/(n+1))]}; shapiro.test(rate$arcsin)
Double-arcsine transformation (PFT)	rate<transform{m1,darcsin=0.5*[asin(sqrt(event/(n+1)))+asin((sqrt(event+1)/(n+1)))]}; shapiro.test(rate$darcsin)
No transformation (PRAW)	rate<-transform[m1, r= event/n]; shapiro.test(rate$r)
Forest plots	forest [meta1, xlim=c(-.1,.8)]
Funnel chart	funnel (meta1)
Egger's test	metabias (meta1, method="linreg")
The sensitivity analysis	Metainf (meta1, pooled = "random") forest (metainf (meta1, pooled = "random"), xlim=c(0, 0.4))
Subgroup analysis	meta1<-metaprop(event, n, study, data=rate, sm="PLN", incr=0.5, allincr=TRUE, addincr=FALSE, title="", byvar= subgroup title, print.byvar=TRUE)
Meta-regression analysis	metareg (meta1, ~covariate title)

Table S4. Included studies and quality scores for pigs, cattle, sheep and goats.

Reference ID	No. tested	No. positive	Prevalence (%)	Random sampling or not 随机采样	Detection method clear or not 检测方法	Detailed sampled method or no T 采样方法	Sample time clear or not 采样时间	Four or more risk factors or not 四个因素	Score	Study Quality
Qi et al. (2017) [31]	400	49	12.25	Y	Y	Y	Y	N	4	High
Wang et al. (2024) [41]	56	1	1.79	Y	Y	Y	Y	N	4	High
Liu et al. (2021) [42]	1063	70	6.59	Y	Y	Y	Y	N	4	High
Fan et al. (2024) [39]	1763	7	0.4	Y	Y	Y	Y	N	4	High
Fu et al. (2021) [43]	204	13	6.37	Y	Y	Y	Y	N	4	High
Qi et al. (2023) [44]	1000	96	9.6	Y	Y	Y	N	N	3	High
Sun et al. (2020) [45]	422	38	9	Y	Y	Y	Y	N	4	High
Liu et al. (2023) [46]	231	24	10.39	Y	Y	Y	Y	N	4	High
Xu et al. (2017) [42]	205	14	6.83	Y	Y	Y	N	N	3	High
Yao et al. (2013) [40]	704	24	3.41	Y	Y	Y	N	N	3	High
Zhang et al. (2010) [33]	60	7	11.7	Y	Y	Y	Y	N	4	High
Sun et al. (2012) [47]	327	20	6.12	Y	Y	Y	Y	N	4	High
Gu et al. (2014) [48]	84	15	17.86	Y	Y	Y	Y	N	4	High
Wu et al. (2020) [49]	360	44	12.22	Y	Y	Y	Y	N	4	High
Zeng et al (2006) [38]	200	50	25	Y	Y	Y	N	N	3	High
Li et al. (2013) [37]	1926	127	6.59	Y	Y	Y	Y	N	4	High
Yang et al. (2020) [50]	302	63	20.86	Y	Y	Y	Y	N	4	High
Wang et al. (2025) [36]	498	11	2.21	Y	Y	Y	Y	N	4	High
Xu et al. (2005) [51]	289	12	4.15	Y	Y	Y	Y	N	4	High
Yu et al. (2024) [52]	50	7	14	Y	Y	Y	Y	N	4	High
Liang et al. (2012) [35]	8773	1132	12.9	Y	Y	Y	Y	N	4	High

Gao et al. (2009) [55]	94	77	81.91	N	Y	Y	N	N	2	Low
Yang et al. (2008) [53]	1032	13	1.26	Y	Y	Y	Y	N	4	High
Lv et al. (2022) [34]	450	57	12.67	Y	Y	Y	Y	N	4	High
Liang et al. (2015) [56]	13728	1505	10.96	Y	Y	Y	N	N	3	High
Peng et al. (2018) [57]	145	4	2.76	N	N	Y	N	N	1	Low
Yu et al. (2025) [54]	36	5	13.89	Y	N	Y	N	N	2	Low
Wang et al. (2021) [58]	90	0	0	Y	Y	Y	Y	N	4	High

Y*: Yes; N*: No.

Table S5. Included studies and quality scores for pigs.

Reference ID	No. tested	No. positive	Prevalence (%)	Random sampling or not 随机采样	Detection method clear or not 检测方法	Detailed sampled method or no T 采样方法	Sample time clear or not 采样时间	Four or more risk factors or not 四个因素	Score	Study Quality
Qi et al. (2017) [31]	400	49	12.25	Y	Y	Y	Y	N	4	High
Liu et al. (2021) [42]	808	70	8.66	Y	Y	Y	Y	N	4	High
Fan et al. (2024) [39]	1763	7	0.4	Y	Y	Y	Y	N	4	High
Fu et al. (2021) [43]	98	6	6.12	Y	Y	Y	Y	N	4	High
Qi et al. (2023) [44]	400	50	12.50	Y	Y	Y	N	N	3	High
Sun et al. (2020) [45]	296	38	12.84	Y	Y	Y	Y	N	4	High
Liu et al. (2023) [46]	231	24	10.39	Y	Y	Y	Y	N	4	High
Yao et al. (2013) [40]	704	24	3.41	Y	Y	Y	N	N	3	High
Zhang et al. (2010) [33]	60	7	11.7	Y	Y	Y	Y	N	4	High
Sun et al. (2012) [47]	145	9	6.21	Y	Y	Y	Y	N	4	High
Gu et al. (2014) [48]	42	4	9.52	Y	Y	Y	Y	N	4	High
Wu et al. (2020) [49]	200	14	7.00	Y	Y	Y	Y	N	4	High
Li et al. (2013) [37]	1110	87	7.84	Y	Y	Y	Y	N	4	High
Yang et al. (2020) [50]	302	63	20.86	Y	Y	Y	Y	N	4	High
Wang et al. (2025) [36]	320	3	0.93	Y	Y	Y	Y	N	4	High
Xu et al. (2005) [51]	98	5	5.10	Y	Y	Y	Y	N	4	High
Yu et al. (2024) [52]	50	7	14	Y	Y	Y	Y	N	4	High
Liang et al. (2012) [35]	8773	1132	12.9	Y	Y	Y	Y	N	4	High
Gao et al. (2009) [55]	94	77	81.91	N	Y	Y	N	N	2	Low
Yang et al. (2008) [53]	360	8	2.22	Y	Y	Y	Y	N	4	High
Lv et al. (2022) [34]	300	24	8.00	Y	Y	Y	Y	N	4	High

Liang et al. (2015) [56]	11403	1472	12.91	Y	Y	Y	N	N	3	High
Peng et al. (2018) [57]	145	4	2.76	N	N	Y	N	N	1	Low
Yu et al. (2025) [54]	36	5	13.89	Y	N	Y	N	N	2	Low

Y*: Yes; N*: No.

Table S6. Included studies and quality scores for cattle.

Reference ID	No. tested	No. positive	Prevalence (%)	Random sampling or not 随机采样	Detection method clear or not 检测方法	Detailed sampled method or no T 采样方法	Sample time clear or not 采样时间	Four or more risk factors or not 四个因素	Score	Study Quality
Wang et al. (2024) [36]	42	1	2.38	Y	Y	Y	Y	N	4	High
Liu et al. (2021) [42]	110	0	0.00	Y	Y	Y	Y	N	4	High
Fu et al. (2021) [43]	64	`4	6.25	Y	Y	Y	Y	N	4	High
Qi et al. (2023) [44]	200	16	8.00	Y	Y	Y	N	N	3	High
Sun et al. (2020) [45]	41	0	0	Y	Y	Y	Y	N	4	High
Xu et al. (2017) [32]	205	14	6.83	Y	Y	Y	N	N	3	High
Sun et al. (2012) [47]	112	3	2.68	Y	Y	Y	Y	N	4	High
Gu et al. (2014) [48]	42	11	26.19	Y	Y	Y	Y	N	4	High
Wu et al. (2020) [49]	160	30	18.75	Y	Y	Y	Y	N	4	High
Li et al. (2013) [37]	485	32	6.60	Y	Y	Y	Y	N	4	High
Wang et al. (2025) [36]	178	8	4.49	Y	Y	Y	Y	N	4	High
Xu et al. (2005) [51]	69	0	0.00	Y	Y	Y	Y	N	4	High
Yang et al. (2008) [53]	288	2	0.69	Y	Y	Y	Y	N	4	High
Lv et al. (2022) [34]	150	33	22.0	Y	Y	Y	Y	N	4	High
Liang et al. (2015) [56]	648	18	2.78	Y	Y	Y	N	N	3	High
Wang et al. (2021) [58]	90	0	0	Y	Y	Y	Y	N	4	High

Y*: Yes; N*: No.

Table S7. Included studies and quality scores for sheep and goats.

Reference ID	No. tested	No. positive	Prevalence (%)	Random sampling or not 随机采样	Detection method clear or not 检测方法	Detailed sampled method or no T 采样方法	Sample time clear or not 采样时间	Four or more risk factors or not 四个因素	Score	Study Quality
Liu et al. (2021) [42]	145	0	0.00	Y	Y	Y	Y	N	4	High
Fu et al. (2021) [43]	42	3	7.14	Y	Y	Y	Y	N	4	High
Qi et al. (2023) [44]	400	30	7.50	Y	Y	Y	N	N	3	High
Sun et al. (2020) [45]	85	0	0.00	Y	Y	Y	Y	N	4	High
Sun et al. (2012) [47]	70	8	11.40	Y	Y	Y	Y	N	4	High
Li et al. (2013) [37]	331	8	2.42	Y	Y	Y	Y	N	4	High
Xu et al. (2005) [51]	122	7	5.70	Y	Y	Y	Y	N	4	High
Yang et al. (2008) [53]	384	3	0.78	Y	Y	Y	Y	N	4	High
Liang et al. (2015) [56]	1677	15	89	Y	Y	Y	N	N	3	High

Y*: Yes; N*: No.

Table S8. Egger's for publication bias

Combine (pigs + cattle + sheep and goats)

Bias	se. bias	t	df	p-value
-1.3640	1.8629	-0.73	26	0.4706

Pigs

Bias	se. bias	t	df	p-value
-2.3429	2.1287	-1.10	23	0.2824

Cattle

Bias	se. bias	t	df	p-value
0.5922	2.1797	0.27	14	0.7898

Table S9. Pooled prevalence of *Y. enterocolitica* in pigs, cattle, sheep and goats in Chinese Mainland.

	No. Studies	No. Tested	No. Positive	% (95% CI*)	Heterogeneity			Univariate meta-regression	
					χ^2	P-value	I^2 (%)	P-value	Coefficient (95% CI)
**Region** [a]									
Eastern	15	5,918	533	9.44% (6.23-13.20)	303.36	< 0.0001	95.4%		
Northeastern	2	724	126	20.64% (0.00-79.67)	256.64	< 0.0001	99.6%		
Northern	1	2,531	388	15.33% (13.95-16.76)	0.00	--	--		
Central	2	3,508	497	13.91% (1.99-33.98)	208.31	< 0.0001	99.5%	0.01	-0.20 (-0.37 to -0.04)
Northwestern	5	3,045	225	9.13% (5.86-13.00)	44.61	< 0.0001	91.0%		
Southern	1	200	50	25.00% (19.23-31.25)	0.00	--	--		
Southwestern	3	4,509	80	1.78% (0.36-4.20)	44.22	< 0.0001	95.5%		
**Study period**									
≤ 2015	7	12,504	1,377	9.69% (4.78-16.04)	316.44	< 0.0001	98.1%		
2016-2020	9	4,503	285	3.74% (1.32-7.23)	245.25	< 0.0001	95.5%	0.03	0.12 (0.00 to 0.23)
≥ 2021	5	1,373	48	3.48% (0.42-8.77)	65.89	< 0.0001	92.4%		
**Sample classification**									
Intestinal contents	3	9,273	1,183	8.68% (2.84-17.17)	17.63	< 0.0001	88.7%		
Meat	10	3,574	233	15.47% (1.99-37.54)	760.45	< 0.0001	98.8%		
Oral contents	4	890	96	12.65% (2.87-27.38)	86.86	< 0.0001	96.5%	0.19	0.12 (-0.06 to 0.30)
Stool	14	20,665	1,973	7.23% (4.74-10.19)	312.62	< 0.0001	95.8%		
Milk	1	90	0.00	0.00% (0.00-1.90)	0.00	--	--		
**Detection methods** [b]									
Culture-based	12	8,825	447	4.60% (2.47-7.30)	323.68	< 0.0001	96.6%		
Pulse-field gel electrophoresis	2	14,055	1,525	8.64% (4.56-13.85)	9.21	0.0024	89.1%		
Loop-mediated isothermal amplification	1	94	77	81.91% (73.42-89.11)	0.00	--	--	0.03	-0.16 (-0.31 to -0.01)
PCR	6	10,470	1,268	8.56% (4.62-13.52)	111.93	< 0.0001	95.5%		
qPCR	2	281	31	10.79% (7.34-14.78)	0.65	0.4216	0.0%		
**Specie**									
Cattle	16	2,884	172	4.67% (1.88-8.47)	165.64	< 0.0001	90.9%	0.01	0.11 (0.02 to 0.20)
Pig	26	28,349	3,239	9.93% (5.79-14.97)	1117.18	< 0.0001	97.8%		

	Sheep and goat	10	3,259	74	1.44% (0.01-4.29)	78.19	< 0.0001	88.5%		
**Quality Points**										
	1-2	3	275	86	28.51% (0-82.49)	210.32	< 0.0001	99.0%		
	3-4	25	34,217	3,399	7.75% (5.41-10.47)	999.38	< 0.0001	97.6%	0.01	-0.28 (-0.50 to -0.06)
**Total**		28	34,492	3,485	9.37% (5.55-14.03)	1262.63	< 0.0001	97.9%		

CI*: Confidence interval

Region[a]: Eastern: Fujian, Shanghai, Jiangsu, Jiangxi, Shandong; Northeastern: Liaoning, Heilongjiang; Northern: Beijing, Inner Mongolia, Tianjin; Northwestern: Ningxia, Qinghai, Shaanxi; Southern: Guangdong; Southwestern: Sichuan, Yunnan; Central: Henan

Detection Methods [b]: PCR: Polymerase Chain Reaction; qPCR: quantitative polymerase chain reaction

Table S10. Pooled prevalence of *Y. enterocolitica* in pigs in Chinese Mainland.

	No. Studies	No. Tested	No. Positive	% (95% CI*)	Heterogeneity			Univariate meta-regression	
					χ^2	P-value	I^2 (%)	P-value	Coefficient (95% CI)
**Region** [a]									
Eastern	14	3528	415	9.89% (7.16-13.66)	129.72	<0.0001	90.0%		
Northeastern	2	546	118	7.19% (0.14-100)	47.69	<0.0001	97.9%		
Northern	1	2531	388	15.33% (13.99-16.80)	0.00	--	--		
Central	2	2692	457	13.59% (4.66-39.68)	94.38	<0.0001	98.9%	0.47	0.27 (-0.48 to 1.04)
Northwestern	4	2690	178	8.42% (5.45-13.02)	30.50	<0.0001	90.2%		
Southern	1	200	50	25.00% (19.66-31.78)	0.00	--	--		
Southwestern	3	4509	80	1.54% (0.43-5.45)	25.62	<0.0001	92.2%		
**Study period**									
≤ 2015	7	13422	1650	8.85% (5.26-14.88)	72.25	<0.0001	91.7%		
2016-2020	8	3174	188	3.61% (1.56-8.36)	67.31	<0.0001	85.1%	0.27	-0.52 (-1.47 to 0.42)
≥ 2021	4	1126	40	3.70% (0.97-14.13)	34.59	<0.0001	88.4%		
**Sample classification**									
Intestinal contents	3	9273	1183	12.84% (12.17-13.54)	7.21	0.0272	72.3%		
Meat	9	2882	143	7.24% (2.58-20.33)	699.59	<0.0001	98.9%	0.99	0.00 (-0.78 to 0.78)
Oral contents	3	869	92	9.26% (2.71-31.65)	82.25	<0.0001	97.6%		
Stool	13	15293	1817	8.09% (5.54-11.80)	118.94	<0.0001	89.9%		
**Detection methods** [b]									
Culture-based	12	6283	421	6.81% (3.68-12.60)	232.54	<0.0001	95.3%		
Pulse-field gel electrophoresis	2	11548	1481	9.61% (4.75-19.43)	5.12	0.0237	80.5%		
Loop-mediated isothermal amplification	1	94	77	81.91% (74.49-90.08)	0.00	--	--	0.34	-0.40 (-1.26 to 0.44)
PCR	6	9951	1220	7.22% (3.69-14.14)	30.95	<0.0001	83.8%		
qPCR	2	281	31	11.14% (7.99-15.52)	0.56	0.4562	0.0%		
**Quality Points**									
1-2	3	275	86	15.35% (2.22-100)	64.23	<0.0001	96.9%		
3-4	22	28063	3153	7.50% (5.13-10.97)	280.99	<0.0001	92.5%	0.20	-0.80 (-2.04 to 0.42)
**Total**	25	28,338	3,239	8.15% (5.44-12.19)	1643.27	0	98.5%		

CI*: Confidence interval

Region[a]: Eastern: Fujian, Shanghai, Jiangsu, Jiangxi, Shandong; Northeastern: Liaoning, Heilongjiang; Northern: Beijing, Inner Mongolia, Tianjin; Northwestern: Ningxia, Qinghai, Shaanxi; Southern: Guangdong; Southwestern:

Sichuan, Yunnan; Central: Henan

Detection Methods [b]: PCR: Polymerase Chain Reaction; qPCR: quantitative polymerase chain reaction.

Table S11. Pooled prevalence of *Y. enterocolitica* in cattle in Chinese Mainland.

	No. Studies	No. Tested	No. Positive	% (95% CI*)	Heterogeneity			Univariate meta-regression	
					χ^2	P-value	I^2 (%)	P-value	Coefficient (95% CI)
**Region [a]**									
Eastern	10	1128	67	4.08% (0.73-9.45)	99.32	<0.0001	90.9%	0.31	-0.09 (-0.26 to 0.08)
Northeastern	1	178	8	4.49% (1.86-8.11)	0.00	--	--		
Central	1	485	32	6.60% (4.55-8.99)	0.00	--	--		
Northwestern	2	355	47	13.44% (2.30-31.40)	17.12	<0.0001	94.2%		
**Study period**									
≤ 2015	5	1017	48	4.28% (0.03-13.38)	48.80	<0.0001	91.8%	0.45	0.07 (-0.11 to 0.26)
2016-2020	6	111	1	6.27% (0.82-15.57)	77.64	<0.0001	93.6%		
≥ 2021	2	703	75	0.52% (0.00-4.61)	1.66	<0.0001	39.7%		
**Sample classification**									
Meat	6	636	87	11.92% (5.08-20.94)	42.68	<0.0001	88.3%	0.02	-0.15 (-0.28 to -0.01)
Stool	9	2158	85	2.45% (0.77-4.88)	50.98	<0.0001	84.3%		
Milk	1	90	0	0.00% (0.00-1.90)	0.00	--	--		
**Detection methods [b]**									
Culture-based	11	1727	110	4.68% (1.34-9.60)	106.36	<0.0001	90.6%	0.95	0.00 (-0.16 to 0.17)
Pulse-field gel electrophoresis	2	760	21	2.64% (1.57-3.95)	0.02	0.8935	0.0%		
PCR	3	397	41	6.19% (0.00-23.11)	42.42	<0.0001	95.3%		
**Quality Points**									
3-4	16	2,884	172	4.67% (1.88-8.47)	165.64	<0.0001	90.9%		
**Total**	16	2,884	172	4.67% (1.88-8.47)	165.64	<0.0001	90.9%		

CI*: Confidence interval

Region[a]: Eastern: Fujian, Shanghai, Jiangsu, Jiangxi, Shandong; Northeastern: Liaoning, Heilongjiang; Northwestern: Ningxia, Qinghai, Shaanxi; Central: Henan

Detection Methods [b]: PCR: Polymerase Chain Reaction

Table S12. Pooled prevalence of *Y. enterocolitica* in sheep and goats in Chinese Mainland.

	No. Studies	No. Tested	No. Positive	% (95% CI*)	Heterogeneity			Univariate meta-regression	
					χ^2	P-value	I^2 (%)	P-value	Coefficient (95% CI)
**Region** [a]									
Eastern	7	1248	51	3.13% (0.51-7.42)	56.29	< 0.0001	89.3%	0.81	0.02 (-0.22 to 0.28)
Central	1	331	8	2.42% (0.99-4.39)	0.00	--	--		
**Study period**									
≤ 2015	3	785	19	3.44% (0.03-10.78)	16.35	0.0003	87.8%		
2016-2020	3	272	3	0.77% (0.00-6.21)	8.48	0.0144	76.4%	0.31	-0.09 (-0.26 to 0.08)
≥ 2021	1	122	7	5.74% (2.19-10.67)	0.00	--	--		
**Sample classification**									
Stool	8	3214	71	2.36% (0.49-5.33)	73.92	< 0.0001	90.5%	0.34	-0.12 (-0.38 to 0.13)
Meat	1	42	3	7.14% (0.93-17.32)	0.00	--	--		
**Detection methods** [b]									
Culture-based	6	1387	44	1.85% (0.12-5.01)	44.11	< 0.0001	88.7%	0.37	
Pulse-field gel electrophoresis	2	1747	23	4.35% (0.00-20.26)	17.86	< 0.0001	94.4%		-0.07 (-0.22 to 0.08)
PCR	1	122	7	5.74% (2.19-10.67)	0.00	--	--		
**Quality Points**									
3-4	9	3256	74	2.62% (0.67-5.57)	77.96	< 0.0001	89.7%		
**Total**	9	3256	74	2.62% (0.67-5.57)	77.96	< 0.0001	89.7%		

CI*: Confidence interval

Region [a]: Eastern: Fujian, Shanghai, Jiangsu, Jiangxi, Shandong; Central: Henan.

Detection Methods [b]: PCR: Polymerase Chain Reaction.

Table S13. Geographical factors affecting the prevalence of *Y. enterocolitica* in pigs, cattle, sheep and goats in Chinese Mainland.

	No. Studies	No. Tested	No. Positive	% (95% CI*)	Heterogeneity			Univariate meta-regression	
					χ²	P-value	I² (%)	P-value	Coefficient (95% CI)
**Longitude**									
≤ 112°	7	8,313	354	5.35% (3.05-8.22)	258.87	< 0.0001	96.1%		
113–117°	11	8,978	1,202	14.66% (8.17-22.61)	956.10	< 0.0001	98.6%	0.02	-0.13 (-0.25 to -0.01)
≥ 118°	8	3,144	343	11.31% (4.49-20.62)	299.23	< 0.0001	97.3%		
**Latitude**									
20-30°	6	3,087	280	14.84% (4.78-29.07)	324.10	< 0.0001	98.1%		
30-40°	18	15,865	1,444	8.70% (5.86-12.02)	1354.66	< 0.0001	98.3%	0.15	-0.09 (-0.22 to 0.03)
40-50°	2	1,483	175	15.13% (0.00-50.41)	269.98	< 0.0001	99.3%		
**Altitude (m)**									
< 1000	14	7,975	901	12.02% (6.80-18.44)	825.60	< 0.0001	98.1%		
1000-10000	7	7,187	727	11.64% (4.07-22.26)	959.43	< 0.0001	99.2%	0.16	-0.09 (-0.23 to 0.04)
> 10000	5	5,273	271	5.99% (3.88-8.50)	65.17	< 0.0001	89.3%		
**Rainfall (mm)**									
< 60	9	10,545	1,097	10.82% (6.33-16.30)	602.24	< 0.0001	97.8%		
60–119.9	10	5,701	531	8.61% (3.53-15.57)	723.59	< 0.0001	98.6%	0.87	0.01 (-0.11 to 0.13)
≥ 120	7	4,189	271	12.23% (4.29-23.30)	488.68	< 0.0001	98.4%		
**Humidity**									
40-55%	4	3,402	425	12.33% (4.43-23.25)	429.93	< 0.0001	98.8%		
55-70%	12	10,390	1,115	11.99% (6.52-18.80)	894.58	< 0.0001	98.2%	0.21	-0.07 (-0.20 to 0.04)
70-85%	10	6,643	359	7.34% (3.53-12.34)	409.43	< 0.0001	97.6%		
**Average annual temperature**									
< 15°C	8	10,135	1,256	11.98% (6.45-18.89)	956.62	< 0.0001	98.6%		
15-17°C	12	9,076	416	6.28% (3.55-9.69)	371.27	< 0.0001	96.5%	0.04	-0.11 (-0.23 to –0.00)
> 17°C	6	1,224	227	17.65% (5.82-33.93)	124.17	< 0.0001	96.0%		
**Climate**									
Plateau and Mountain climate	4	2,595	168	7.65% (4.87-10.96)	27.86	< 0.0001	85.6%		
Subtropical Monsoon climate	12	7,893	510	10.10% (5.00-16.68)	639.96	< 0.0001	97.8%	0.58	-0.04 (-0.21 to 0.12)
Temperate Monsoon climate	10	9,947	1,221	11.69% (5.90-19.06)	1081.27	< 0.0001	98.8%		

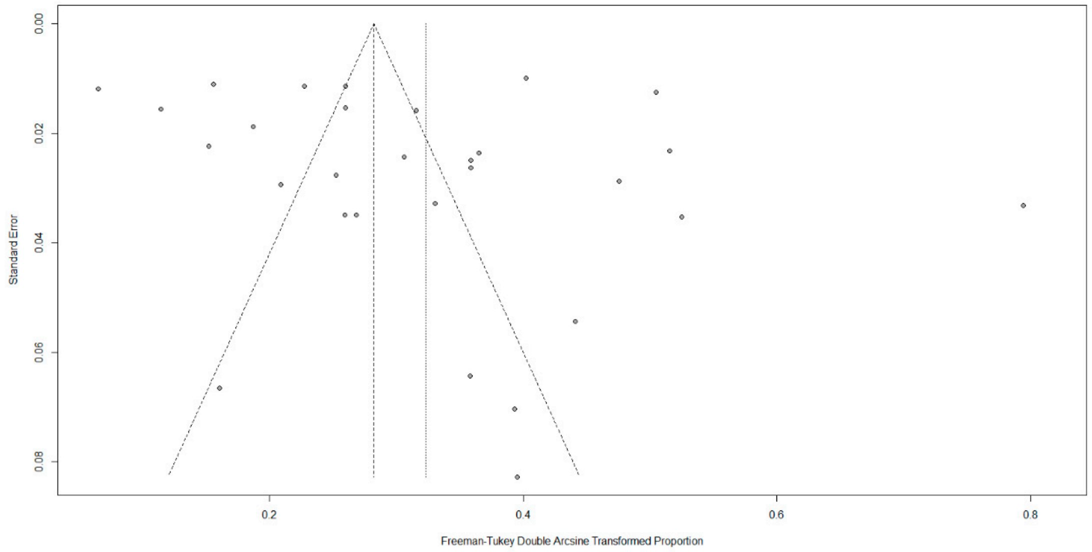

Figure S1. Funnel plot with pseudo 95% confidence limit intervals for the examination of publication bias in the region subgroup of pigs, cattle, sheep and goats.

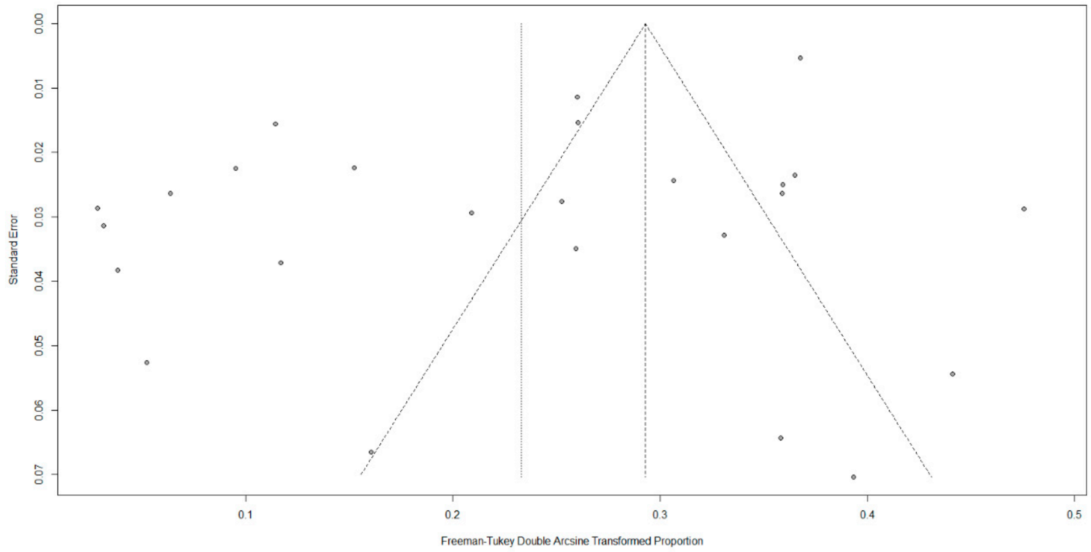

Figure S2. Funnel plot with pseudo 95% confidence limit intervals for the examination of publication bias in the study period subgroup of pigs, cattle, sheep and goats.

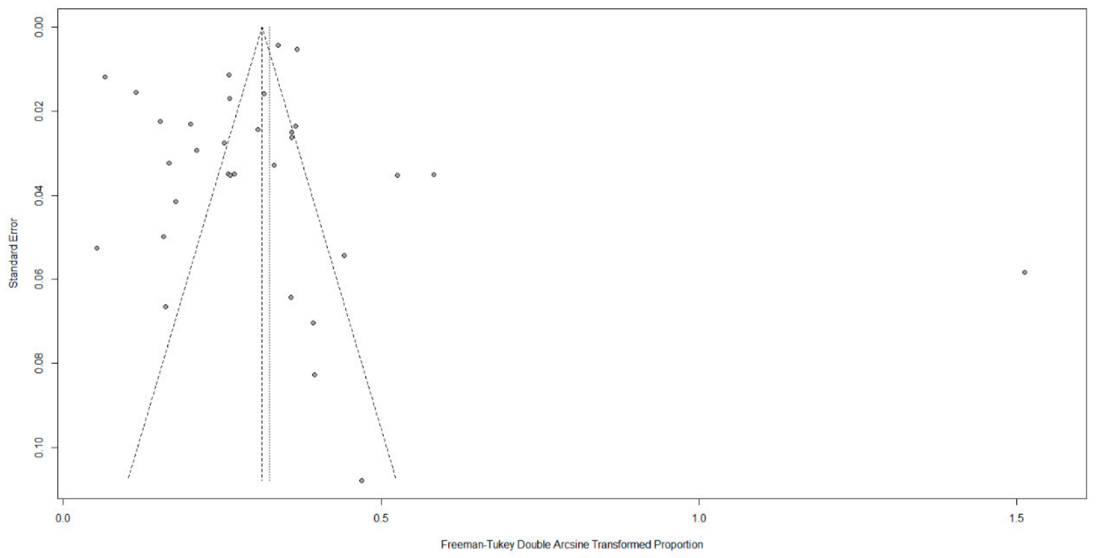

Figure S3. Funnel plot with pseudo 95% confidence limit intervals for the examination of publication bias in the sample classification subgroup of pigs, cattle, sheep and goats.

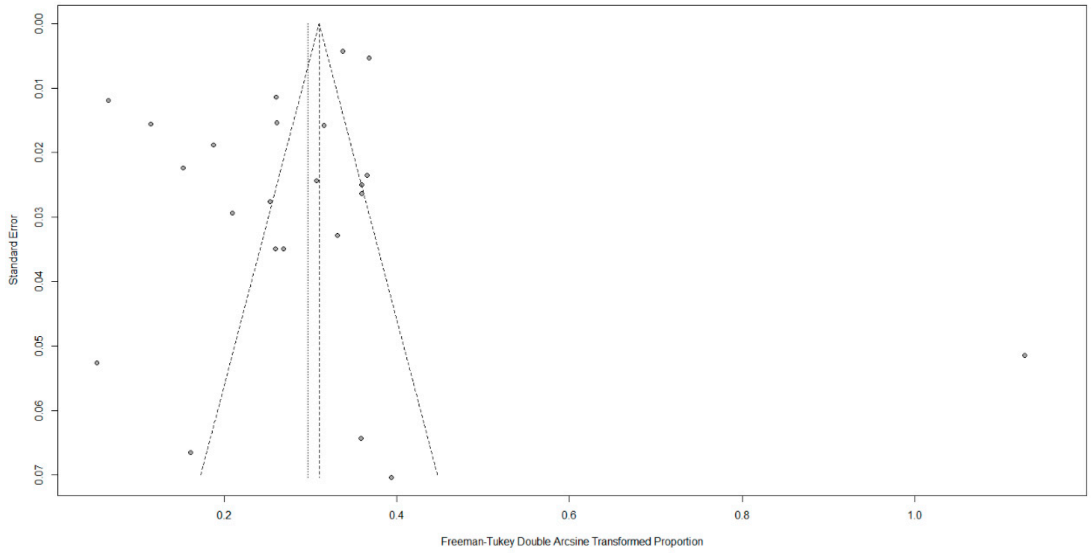

Figure S4. Funnel plot with pseudo 95% confidence limit intervals for the examination of publication bias in the detection method subgroup of pigs, cattle, sheep and goats.

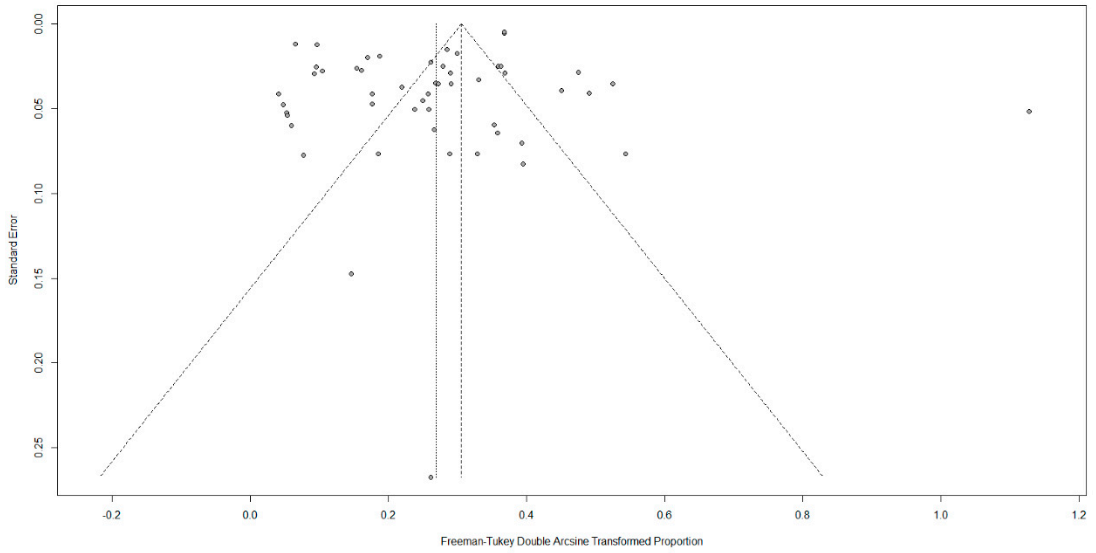

Figure S5. Funnel plot with pseudo 95% confidence limit intervals for the examination of publication bias in the specie subgroup of pigs, cattle, sheep and goats.

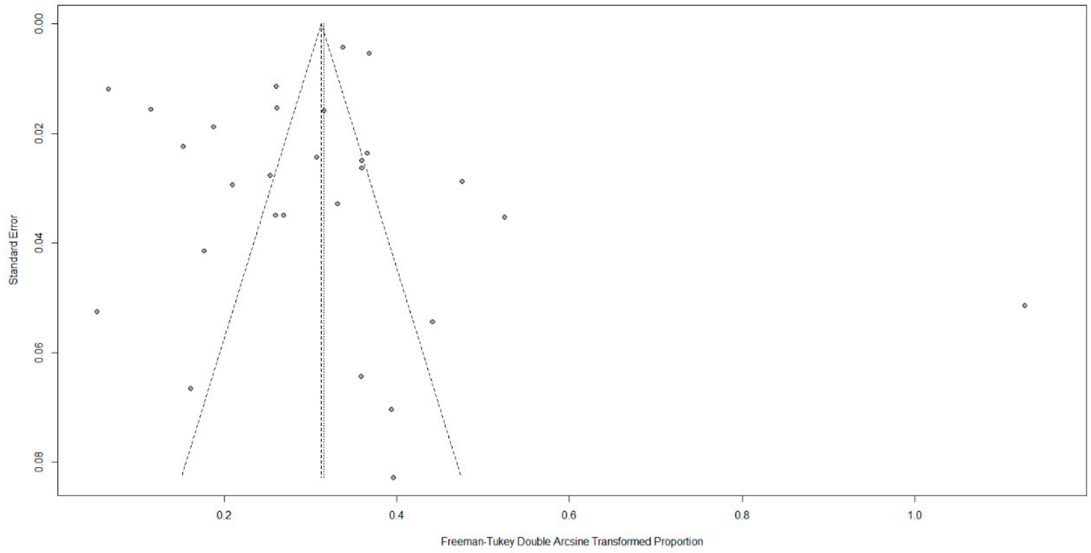

Figure S6. Funnel plot with pseudo 95% confidence limit intervals for the examination of publication bias in the quality points subgroup of pigs, cattle, sheep and goats.

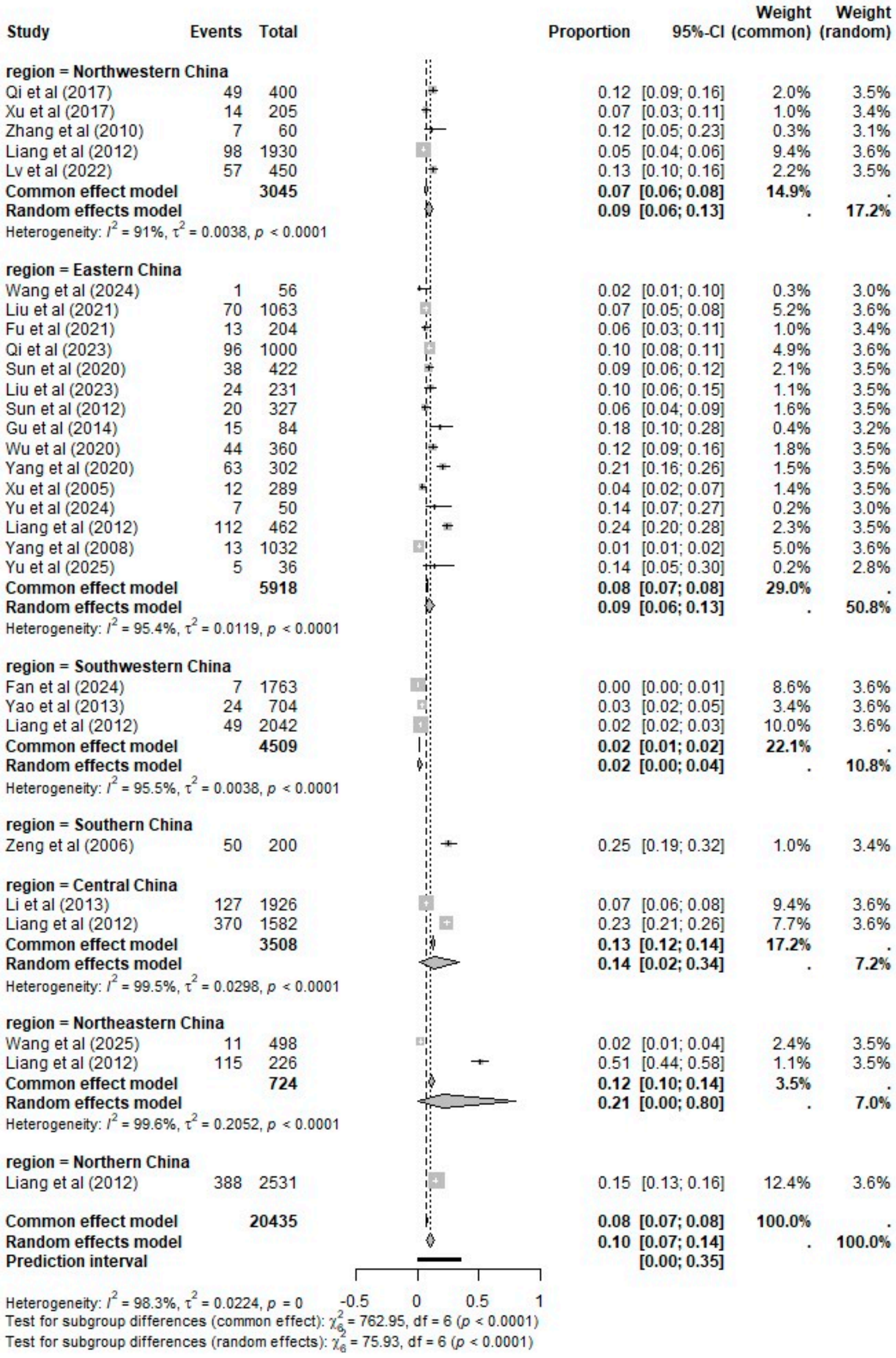

Figure S7. Forest plot of the region subgroup of pigs, cattle, sheep and goats.

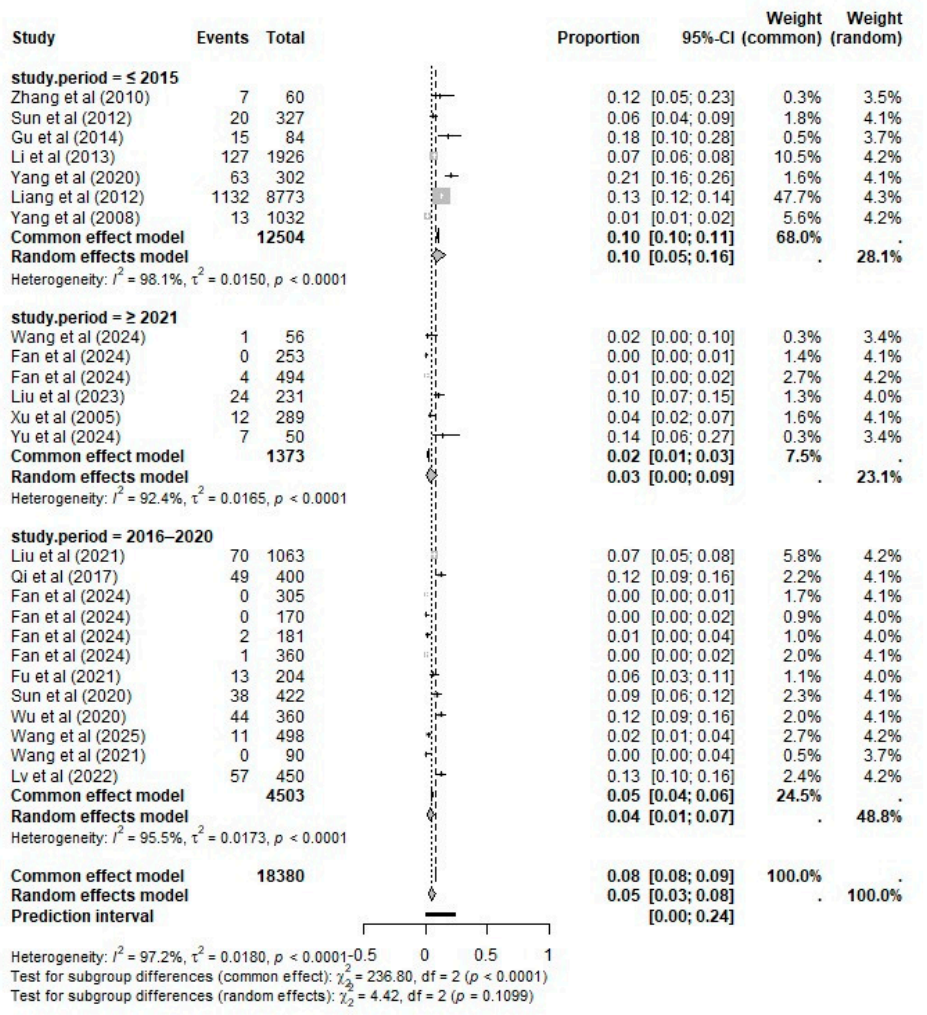

Figure S8. Forest plot of the study period subgroup of pigs, cattle, sheep and goats.

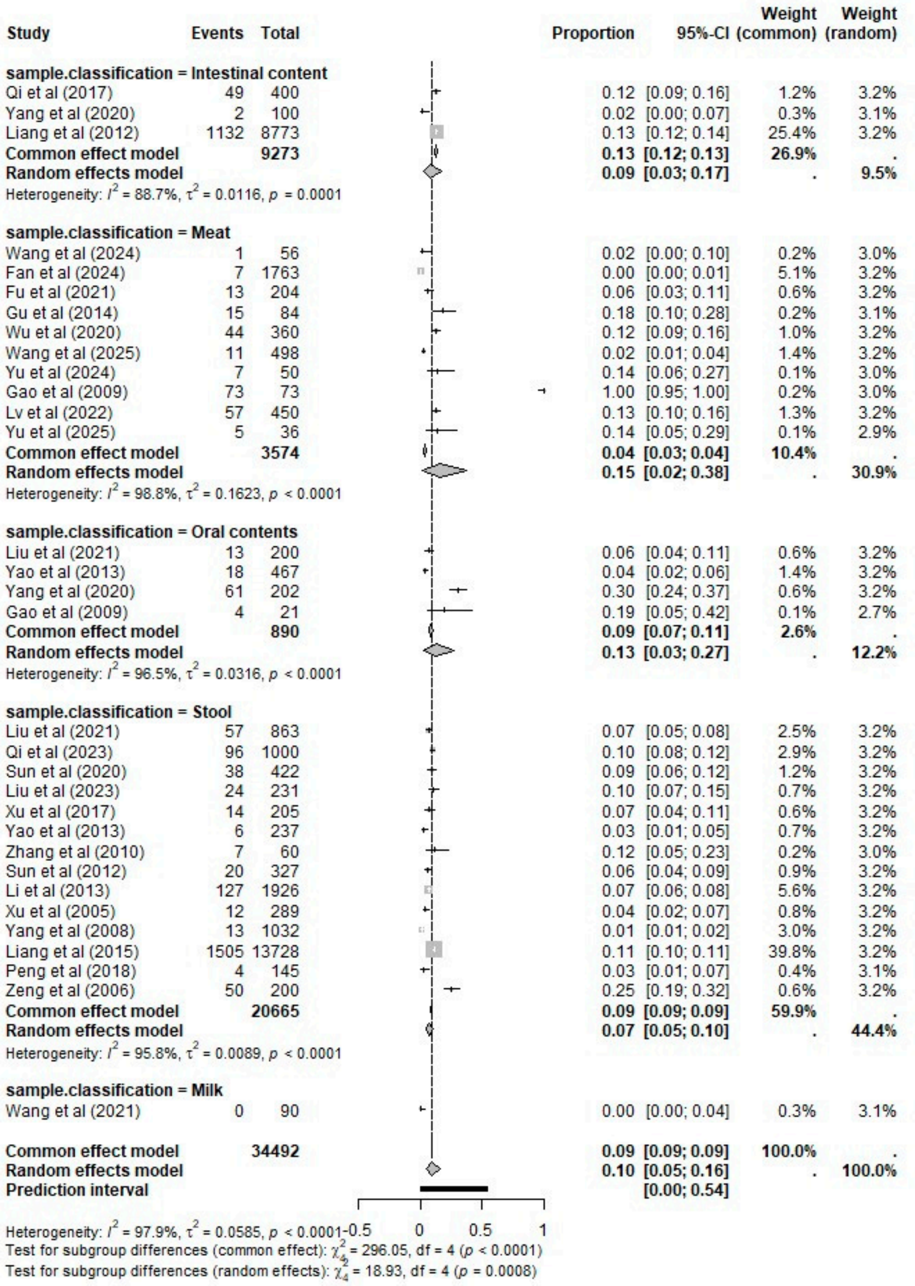

Figure S9. Forest plot of the sample classification subgroup of pigs, cattle, sheep and goats.

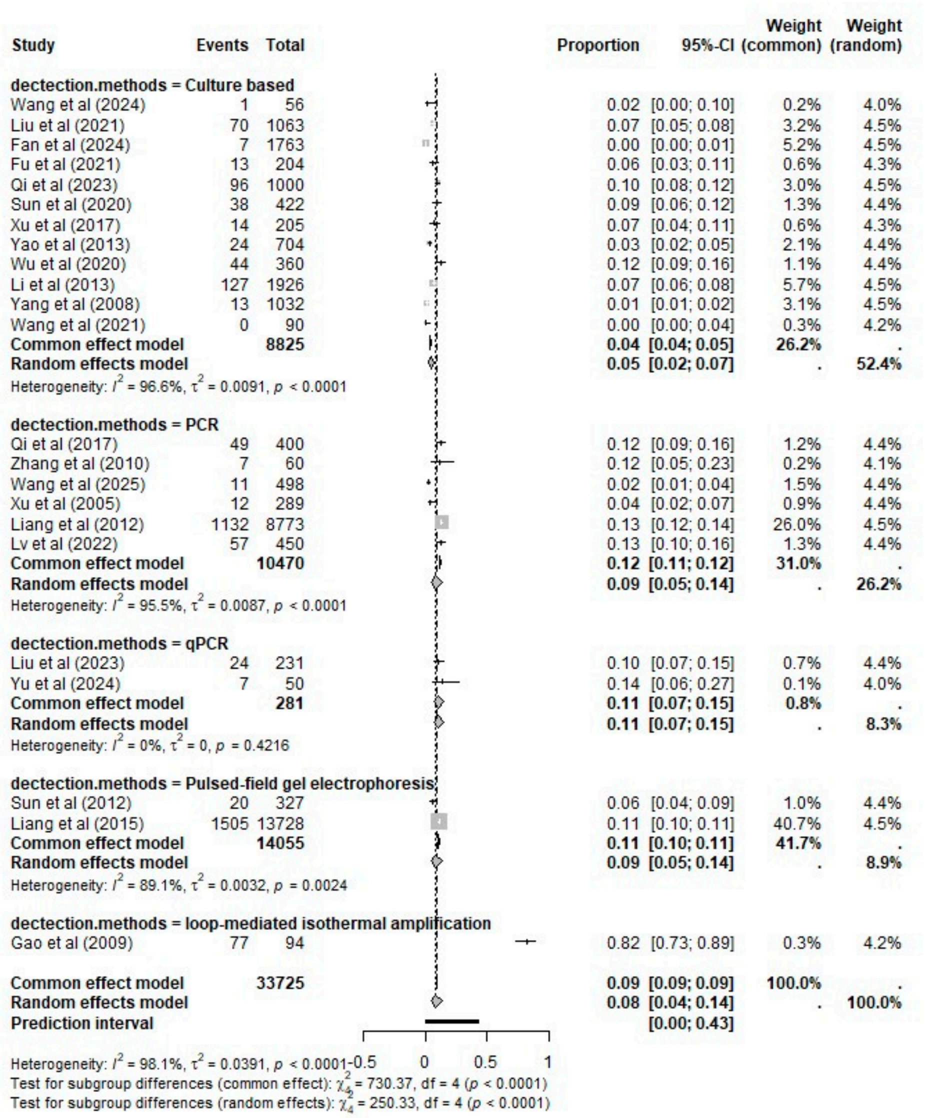

Figure S10. Forest plot of the detection methods subgroup of pigs, cattle, sheep and goats.

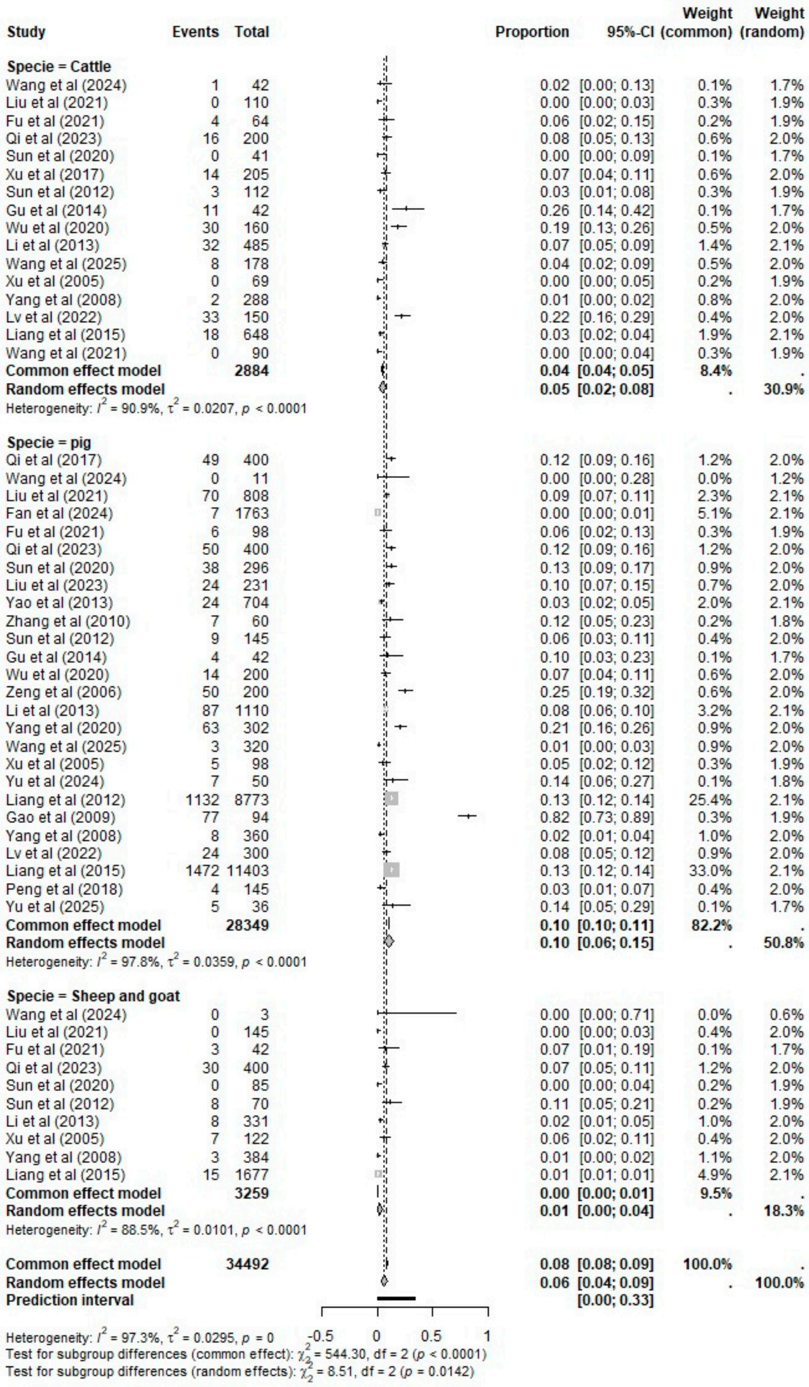

Figure S11. Forest plot with of the specie subgroup of pigs, cattle, sheep and goats.

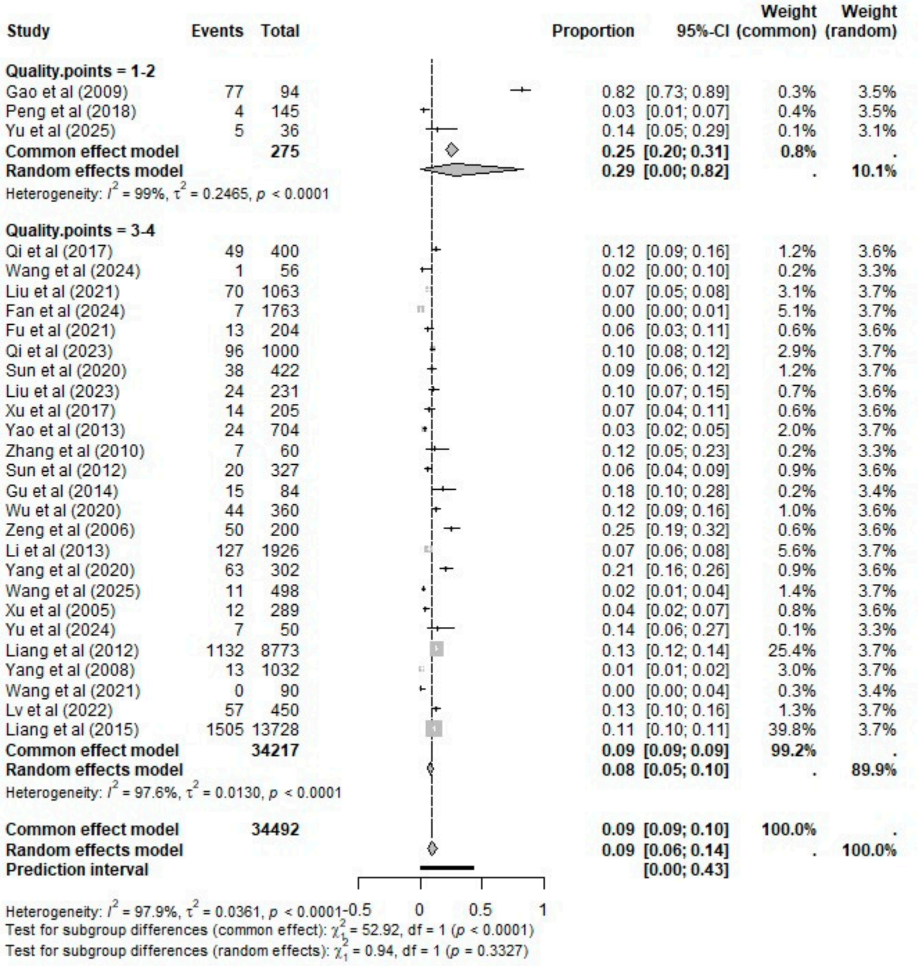

Figure S12. Forest plot of the quality points subgroup of pigs, cattle, sheep and goats.
